# How best to assess quality of life in informal carers of people with dementia; A systematic review of existing outcome measures

**DOI:** 10.1371/journal.pone.0193398

**Published:** 2018-03-14

**Authors:** Johanne Dow, Jonah Robinson, Shannon Robalino, Tracy Finch, Elaine McColl, Louise Robinson

**Affiliations:** 1 Institute of Health & Society, Newcastle University, Newcastle, United Kingdom; 2 Barts and the London School of Medicine and Dentistry, Queen Mary University of London, London, United Kingdom; University of Exeter, UNITED KINGDOM

## Abstract

**Background:**

In the UK, there are currently 800 000 people living with dementia. This number is expected to double in the next 20 years. Two-thirds of people with dementia live in the community supported by informal carers. Caring for a person with dementia has adverse effects on psychological, physical, social wellbeing and quality of life. The measurement of quality of life of carers of people with dementia is increasingly of interest to health and social care practitioners and commissioners, policymakers, and carers themselves. However, there is lack of consensus on the most suitable instrument(s) for undertaking this.

**Methods:**

A systematic review of the literature using COSMIN methodology. Searching of electronic databases (Medline, PsycINFO, CINAHL and Web of Science), reference list and citation searching of key papers was undertaken. COSMIN methodology was used to simultaneously extract data from and assess methodological quality of included studies, and make a recommendation for the instrument with the most high quality evidence for its measurement properties.

**Results:**

Ten instruments were suitable for inclusion in this review. The Carer well-being and support questionnaire (CWS) has the best quality evidence for the greatest number of measurement of properties. The Caregiver Well-Being Scale is also worthy of consideration. There is not presently a measure which could be recommended for use in economic evaluations, however the Impact of Alzheimer’s Disease on the Caregiver questionnaire (IADCQ) could potentially be used following further investigation of its measurement properties in a representative population.

**Conclusion:**

The CWS is the most appropriate instrument to recommend for the assessment of quality of life in informal carers of people with dementia at present. All instruments included in this review would benefit from more rigorous evaluation of their measurement properties.

## Introduction

Dementia is the 9^th^ most burdensome illness globally in terms of disability-adjusted life years (DALYs) [1]. In 2015, 46.8 million people were estimated to be living with dementia [2] with the cost of ‘care’ (direct costs of medical/social care plus the costs of informal care from friends and families) estimated to be $818 billion [1] worldwide. In the United Kingdom (UK), the annual cost of dementia is estimated at £26.3 billion with informal care accounting for nearly half of this total [3]. Globally, policy recommendations focus on improving care and services for both people with dementia and their family carers [1, 4]. Informal caregiving has considerable negative effects on a person’s physical, psychological, financial and social wellbeing [4, 5]. The Royal College of General Practitioners (RCGP) define a carer as: *“A person of any age…who provides unpaid support to a partner, child, relative or friend who couldn't manage to live independently or whose health or wellbeing would deteriorate without this help. This could be due to frailty, disability or serious health condition, mental ill health or substance misuse.”[6]*

A meta-analysis of 84 articles comparing caregivers and non-caregivers observed statistically significant differences between the groups in measures of depression, stress, subjective wellbeing and physical health [7]. Carers of people with dementia are particularly susceptible to the negative impacts of caregiving compared to carers of physically impaired older people, reporting more stress and mental health problems, less time for other family members and more work-related difficulties [8–10].

Considerable research has focused on developing and evaluating a wide range of behavioural and supportive interventions for informal carers of people with dementia [11, 12] with a view to improving their health and wellbeing; however many intervention studies have used an equally broad range of outcome measures to determine their effectiveness [11]. Such outcomes have included: psychological and mental morbidity (including guilt, anxiety, stress, depression and burden), physical health, appraisal of role performance, self-efficacy, coping skills, carers knowledge of dementia, quality of life and symptoms of the person with dementia, health care utilisation by the person with dementia (admission to residential care and number of GP visits) and measures of healthcare expenditure [11]. The use of such a wide range of both interventions and measures to assess effectiveness makes comparison between them difficult [11, 12]. From the user perspective, carers of people with dementia prioritise interventions which increase their quality of life and the information and support they receive [13]. A 2008 pan-European consensus agreed key domains on which psychosocial interventions for people with dementia and their carers should focus, and identified appropriate outcome measures for their effects [14]. For carers, these included mood, burden and quality of life [14].

Quality of life measurement can measure the concept as a whole, or more specific aspects, such as health-related or disease-specific quality of life. Evaluating quality of life broadly is recommended when assessing conditions or interventions which affect the individual as a whole, and their ability to function in multiple roles within their family and workplace [15]. Health-related quality of life measurement places more emphasis on physical and mental functioning, focusing only on the areas of life which would be expected to be affected by a health condition or treatment[15, 16]. Whether measuring quality of life or health-related quality of life, instruments can be generic, disease or symptom specific [16]. Generic instruments allow comparison between populations with different health profiles and conditions [16]; instruments in common use include WHOQOL [17], the EuroQOL (EQ5D) [18] and short-form health survey questionnaires (such as the SF-36) [19]. In measuring quality of life of informal carers of people with dementia, generic instruments have been criticised for lacking validity [14, 20]. The dimensions of generic instruments are also criticised for being insensitive to the psychological consequences and the positive aspects of providing care [21].

Quality of life instruments also differ in how they are scored. “Descriptive” instruments contain multiple domains, and generate a separate score for each domain [22]. Other instruments produce an “index” measurement, which combines the scores from all domains into a single metric figure [22]. Only index measurements can be used to compare quality of life between groups in economic evaluations; which is an important consideration if evidence of cost-effectiveness of an intervention is required for decision-making by service commissioners [22].

In their 2008 pan-European consensus, Moniz-Cook et al. noted the paucity of studies investigating the measurement properties of generic instruments including the WHOQOL, EQ5D, SF-12 and SF-36 in carers of people with dementia, and concluded that the measurement of quality of life of carers of people with dementia was “in its infancy.”[14]. A recent systematic review and consensus conference recommended that the DEMQOL (health-related quality of life in dementia) instrument[23] should be used to measure quality of life of people with dementia, but did not recommend an instrument for carers [24]. A systematic review of disease-specific instruments measuring quality of life of family carers of people with neurodegenerative diseases [25] found that for carers of people with dementia, the Caregiver Quality of Life (CGQOL) Instrument[26] had the most robust psychometric evidence. However, this review did not explore the psychometric properties of generic instruments in this population. There is therefore currently a lack of evidence on the acceptability and psychometric properties of such instruments. The aim of this systematic review was to identify and determine the measurement properties of instruments (generic and disease-specific) which measure quality of life in informal carers of people with dementia in order to identify the most appropriate measure(s) for use in future research.

## Methods

The Consensus-based Standards for the selection of health status measurement instruments in medicine (COSMIN) methodology was used to conduct this systematic review. COSMIN was developed by an international expert panel to evaluate the methodological quality of studies on the measurement properties of health-related patient-reported outcomes (HR-PROS) [27]. Consensus was established on which measurement properties are important, their most adequate definitions and how they should be assessed [28]. The COSMIN checklist was used to simultaneously assess methodological quality [27] and extract data from included studies [29]. A protocol for this study was peer-reviewed in our department and attached as a supplementary file (S1 File).

### Study characteristics

#### Construct of interest

The constructs of interest in this review were quality of life (QoL) and health-related quality of life (HRQoL). Quality of Life is a broad ranging, multidimensional concept which encompasses all aspects of life. It is defined by the WHO as *“individual’s perception of their position in life in the context of the culture and value systems in which they live and in relation to their goals, expectations, standards and concerns” [30]*. It is affected by personal factors including physical and psychological health, social relationships, personal beliefs and self-sufficiency [30], and environmental factors such as finance, job satisfaction and family circumstances. Health-related quality of life is also a multidimensional construct, which encompasses physical, social and psychological health in relation to a health status or condition [28].

#### Inclusion/exclusion criteria

These are shown in Table 1. The criteria were piloted by two researchers (JD and JR) on a sample of 200 search results before being used to screen all titles and abstracts independently.

**Table 1:** Inclusion/exclusion criteria and search strategy.

Inclusion Criteria	• Instruments based on a conceptual framework created to assess QoL or HRQoL • Study population includes informal carers of people with dementia as part or whole of the study population • Self-report instruments, where status is reported directly by an individual without interpretation of another • Studies reporting development of an instrument, or establishment of one or more of its measurement properties as per COSMIN taxonomy
Exclusion criteria	• Studies published in a language other than English • Studies which report only on the use or application of an instrument, without establishment of its psychometric properties. • Instruments which measure the status of the person with dementia only, or whole family or carer-care recipient dyad only • Instruments which seek factual information only without appraisal of these by carer with reference to QoL or HRQoL.
Search Strategy	• Electronic searches: MEDLINE (through OVID)(1946 – April 2016); PsycINFO (1967 – April 2016); CINAHL (1981 – April 2016); Web of Science (1946 – April 2016). Searches updated June 2016. • Additional literature searches: reference lists of articles selected for inclusion were examined to search for additional relevant studies [28]. Citation searching of 3 key papers was also undertaken [26, 31, 32], and 6 authors were contacted for information.
Search terms	• Key words and MeSH headings developed for dementia, quality of life and informal carers. These were combined with each other and then with the methodological filter[33]. A sample search strategy for MEDLINE is available (S2 File)

### Search strategy

The search strategy is summarised in Table 1. The full strategy is available in a supplementary file (S2 File).

#### Electronic search strategy

Studies on measurement properties can be difficult to find, due to variation in terminology, unpredictable or incomplete indexing, and poor reporting in abstracts [28, 33]. The assistance of information specialists (AI and SR) was therefore sought. Two search filters have been developed for improving identification of studies on measurement properties in MEDLINE through PubMed; both have been validated in MEDLINE though PubMed [33]. The highly sensitive filter was selected for use in this review, and was adapted for use in MEDLINE using OVID (by SR), CINAHL, PsycINFO and Web of Science (by JD with review by SR).

#### Study selection

In managing results of the literature search, the Preferred Reporting Items for Systematic Reviews and Meta-Analyses (PRISMA) statement [34] was referred to, and references managed using software (EndNote X7).

Inclusion and exclusion criteria were applied independently by 2 researchers (JR, JD) to the titles and abstracts of articles identified by the literature search. If there was doubt regarding the eligibility of a study, the full paper was obtained for screening. Duplicate studies were removed. Full papers were screened by JD. A list of papers excluded from the review was maintained on bibliographic software, and the reasons for exclusion documented (S3 File). The search process is documented in a flow chart, as recommended by the PRISMA statement [34] and COSMIN method [28].

#### Data extraction and analysis

Data extraction was undertaken by one researcher (JD), following pilot data extraction from one paper selected for inclusion in the review by 2 researchers (JD and EM). The COSMIN checklist with 4-point scale [29] and a second form for data relevant to this review were used for data extraction. The COSMIN manual [29] was referred to throughout the process of data extraction; COSMIN definitions for measurement properties were used (see Table 2) [28]. The checklist was completed for each measurement property tested in each study, in case different samples of the same study population were used for testing each measurement property [29].

**Table 2:** Definitions of measurement properties.

Measurement property	Definition
Reliability	Freedom from measurement error with repeated measurement: • inter- and intra-rater reliability • test-retest reliability • internal consistency e.g. using different sets of items from the same multi-item measurement instrument
Measurement error	*“The systematic and random error of a patient's score that is not attributed to true changes in the construct to be measured”[27]*
Content validity	The adequacy of the instrument in measuring the construct under study
Structural validity	*“The degree to which the scores of a measurement instrument are an adequate reflection of the dimensionality of the construct to be measured”[27]*
Hypotheses testing	Undertaken to investigate construct validity: the extent to which an instrument’s scores are consistent with a priori hypotheses regarding expected mean differences between groups, expected correlations with scores on another instrument or with demographic or clinical variables
Criterion validity	There are no “gold standards” for health-related patient-reported outcomes, therefore criterion validity can only be established when a short version of an instrument is compared to its original long version.[29]
Cross-cultural validity	*“The degree to which the performance of the items on a translated or culturally adapted PRO instrument are an adequate reflection of the performance of the items in the original version of the instrument”[27]*
Responsiveness	*“The ability of the instrument to detect change over time in the construct to be measured”[27]*

The characteristics of each instrument identified, the characteristics of the populations of included studies and the methodological quality of included studies were compared (Table 3, Table 4, S4 and S5 Files). “Best evidence” synthesis was performed by considering the methodological quality of the studies, the consistency of the results and the homogeneity of the studies [28]. The “level of evidence” for each property of each instrument was used to compare the instruments included in the study. The criteria for each level of evidence is shown in Table 5 [28].

**Table 3:** Characteristics of included instruments and summary of quality assessment.

Instrument (and version)	Construct and Domains	1. Construct/Instrument Development 2. Target population	Number of: i. scales ii. items iii. response categories	Scoring: i. index or descriptive ii. range	Administration i. mode ii. time	Instructions for completion	Summary of quality assessment for measurement properties
Caregiver-targeted quality-of-life measure (CGQOL) (Vickrey et al. 2009)	• CGQOL • 10 dimensions: caregiving assistance in ADLS, caregiving assistance in IADLS, personal time, role limitations due to caregiving, family involvement, demands of caregiving, worry, caregiver feelings, spirituality and faith, benefits of caregiving	1. Focus groups (n = 6) and cognitive interviews (n = 29) with carers of people with dementia. 2. Informal carers of people with dementia	i. 10 ii. 80 iii. 5	i. Descriptive ii. 0–100	i. Telephone interview ii. Median 23.5 minutes	unknown	1 study: fair (internal consistency, reliability and structural validity), excellent (hypotheses testing)
Carer well-being and support questionnaire (CWS) (Quirk et al. 2012)	Carer Well-being (10 domains): your day-to-day life, your relationship with the person you care for, your relationships with family and friends, your financial situation, your physical health, your emotional wellbeing, stigma and discrimination, your own safety, the safety of the person you care for, your role as a carer. Carer Support scale (5 domains): information and advice for carers, your involvement in treatment and care planning, support from medical and/or care staff, support from other carers, taking a break (respite)	1. Psychometric analysis of pre-existing instrument (CUES-C) and workshops with informal carers of people with mental health conditions including dementia 2. Informal carers of people with mental health problems including dementia	i. 2 ii. CWS-v1: 74 (43 wellbeing, 31 support); CWS-v2: 49 iii. Wellbeing: 5, Support:4	i. Descriptive ii. unknown	i. Self-administered ii. unknown	written	1 study: CWS-v2: Excellent (internal consistency, content validity, structural validity, hypotheses testing), good (reliability)
Impact of Alzheimer’s Disease on Caregiver Questionnaire (Cole et al. 2014)	Impacts of caregiving on HRQoL. 6 domains: emotional, physical, social, time, sleep and financial	1. Draft instrument developed from systematic review; refined following focus group (21 informal carers of people with Alzheimer’s disease). 2. Informal carers of people with Alzheimer’s Disease	i. 1 ii. 12 iii. 5	i. Index ii. 0–48	i. Internet-based self-administered survey ii. unknown	written	1 study: Excellent (internal consistency), good (reliability), fair (hypotheses testing)
Medical Outcomes Study Short-Form Health Survey (SF-36) (Machniki et al. 2009)	Health-related Quality of Life (HRQoL) Physical health subscales: physical functioning, role limitations due to physical health, bodily pain, general health perceptions Mental health subscales: vitality, social functioning, role limitations due to emotional problems, general mental health.	1. SF-20 instrument revised. Items added to domains for physical functioning, role functioning, bodily pain, social functioning and general health perception. Response choices for physical function revised. Items added to distinguish between role limitations due to physical and mental health problems. 5-item scale for general health perception revised. 2. General population	i. 8 ii. 36 iii. 3	i. descriptive ii. unknown	i. Self-administered questionnaire ii. unknown	written	1 study: Good (internal consistency, structural validity), fair (hypotheses testing)
Caregiver Well-Being Scale (first version- refined by Rubio, Berg-Weber & Tebb 1999)	Compared to Tebb 1995 –factors in basic human needs reduced to: love, physical needs and self-security, and activities of daily living reduced to time for self, household maintenance and family	1. As per first version (Tebb 1995) 2. All informal caregivers	i. 2 ii. 42 (3 items from basic human needs factor in Tebb’s 1995 questionnaire deleted due to poor reliability and validity) iii. 5	i. descriptive ii. unknown	i. Self-administered questionnaire ii. unknown	unknown	
Caregiver Well-Being Scale (second version–Rubio et al. 2003)	Tebb’s 1995 version revised to 2 dimensions “Needs” and “Activities”. Based on Maslow’s (1962) Hierarchy of Needs: lower level needs (physiological needs must be met before higher level needs including (in order): need for safety, love and belongingness, self-esteem and self-actualisation.	1. Items on original scale revised using Maslow’s (1962) hierarchy of needs. Content validity assessed by expert panel comprising 6 professionals and 6 lay experts (family caregivers of people with dementia). 2. Informal caregivers	i. 2 ii. 18 iii. 5	i. descriptive ii. unknown	Not applicable: assessment of content validity by expert panel	n/a	
Caregiver Well-Being Scale: Short-Form Rapid Assessment (Tebb 2013)	Tebb’s 1995 version revised to 2 dimensions “Needs” and “Activities”. Based on Maslow’s (1962) Hierarchy of Needs: lower level needs (physiological needs must be met before higher level needs including (in order): need for safety, love and belongingness, self-esteem and self-actualisation.	1. Original version revised using Maslow’s (1962) Hierarchy of Needs and results of earlier studies on its psychometric properties. Subscales renamed. Content validity assessed by expert panel (5 psychometricians and 1 social worker) and lay panel (10 family caregivers of people with Alzheimer’s disease): 11 items reworded and 1 item deleted. 2. Informal caregivers	i. 2 ii. 16	descriptive	i. Self-administered questionnaire ii. unknown	unknown	

**Table 4:** Characteristics of included study populations.

Instrument, (Country of Study)	Language of Instrument	Sample a. size b. % female	Proportion of dementia carers in sample	Age of carer (mean and range)	Age of care recipient (mean and range)	Relationship of carer and care recipient	Carer living with care recipient	Duration of caring	Hours caring per week
CGQOL (USA) Vickrey et al. 2009	English, Spanish	i. 200 ii. 79%	100%	61.5 (SD 13.5)	80.2 (SD 10)	Spouse 45%, Child/child-in-law 43%, Sib/sib-in-law 4%, Niece/nephew 1%	unclear	42% > 5 years, 21% 3–5 years, 14% 2–3 years, 14% 1–2 years, < 1yr 11%	0–5 hours 9%, 6–10 hours 12%, 11–20 hours 13%, 21–30 hours 10%, >30 hours 57%
CWS (UK) Quirk et al. 2012	English	i. Phase 1: 23, Phase 2: 210, Phase 3: 361 ii. Ph2 72% iii. Ph3 65.3%	i. Phase 1: 8/23 = 34.7% ii. Phase 2 and 3:unknown; samples were carers of people with dementia or mental health problems	i. Phase 1 unknown ii. Phase 2 65.3 (SD 13.4) iii. Phase 3 65.5 (SD 13.1)	unknown	i. phase 1: unknown ii. Phase 2: partner/spouse 44.8%, son/daughter 13.3%, brother/sister 2.4%, parent 35%, friend 1.4%, other 3.3% iii. Phase 3: partner/spouse 54%, son/daughter 32%, brother/sister 1.66%, parent 15.2%, friend 1.7%, other 2.2%	unknown	unknown	unknown
IADCQ (USA) Cole et al.2014)	English	i. 200 ii. 60%	100% (carers of people with Alzheimer’s disease only)	2% ≥ 70; 47% 50–69; 39.5% 30–49; 11% 18–29	unknown	unknown	unknown	< 6 months10%, 6–12 months 22.5%, 13–24 months 25.5%, >2y 42%	unknown
SF-36 (Argentina)Machniki et al. 2009	Spanish	i. 52 ii. 85.4%	100% carers of people with Alzheimer’s disease	58.8±14.9 yrs (66.7% 29–65, 33.3% 66–89)	74.7±7.4	Spouse 50%, children 37.5%, other 12.5%	Yes 75%	unknown	mean 33.7±18.3 (range 6–56)
Caregiver Well-Being Scale (USA) Tebb 1995, Rubio, Berg-Weber & Tebb 1999, Rubio et al 2003, Tebb et al. 2013.	English	Tebb 1995, Rubio, Berg-Weber & Tebb 1999 i. 165 ii. 70%	27/165 = 16.4% Also included: 77 (46%) non-carers, 8 (5%) carers of children with severe developmental disabilities and 53 (32%) carers of “healthy” children <12 years old.	unknown	unknown	unknown	unknown	unknown	unknown
		Rubio et al 2003: i. 12 ii. unknown	50% - 6 lay experts on expert panel for content validity analysis (others were 5 academics engaged in research on family caregiving and one expert who worked with family caregivers)	unknown	unknown	unknown	unknown	unknown	unknown
		Tebb et al. 2013: i. 493 ii. 96%	Informal carers from 3 other study samples: 1. 378 nurses, also informal carers 2. 100 carers of relatives with physical and/or cognitive impairment 3. 15 carers of relatives with dementia	Sample 1: mean age 52, SD 4.9, range 41 -65Samples 2 and 3: unknown	unknown	Sample 1: 77% daughter, 12% daughter-in-law. Samples 2 and 3: unknown	unknown	unknown	sample 1: average 6 hrs/week, samples 2 and 3 unknown

#### Quality assessment

The methodological quality of included studies was assessed by scoring the quality of the study for each measurement property examined using the COSMIN checklist with 4-point scale. A methodological quality score per property was obtained using the lowest rating of any item on the checklist (“worst score counts”) [29]. It was not possible to assess the impact of publication bias on the studies included in this review, as there is currently no register of studies on measurement properties [28].

## Results

**Fig 1:**
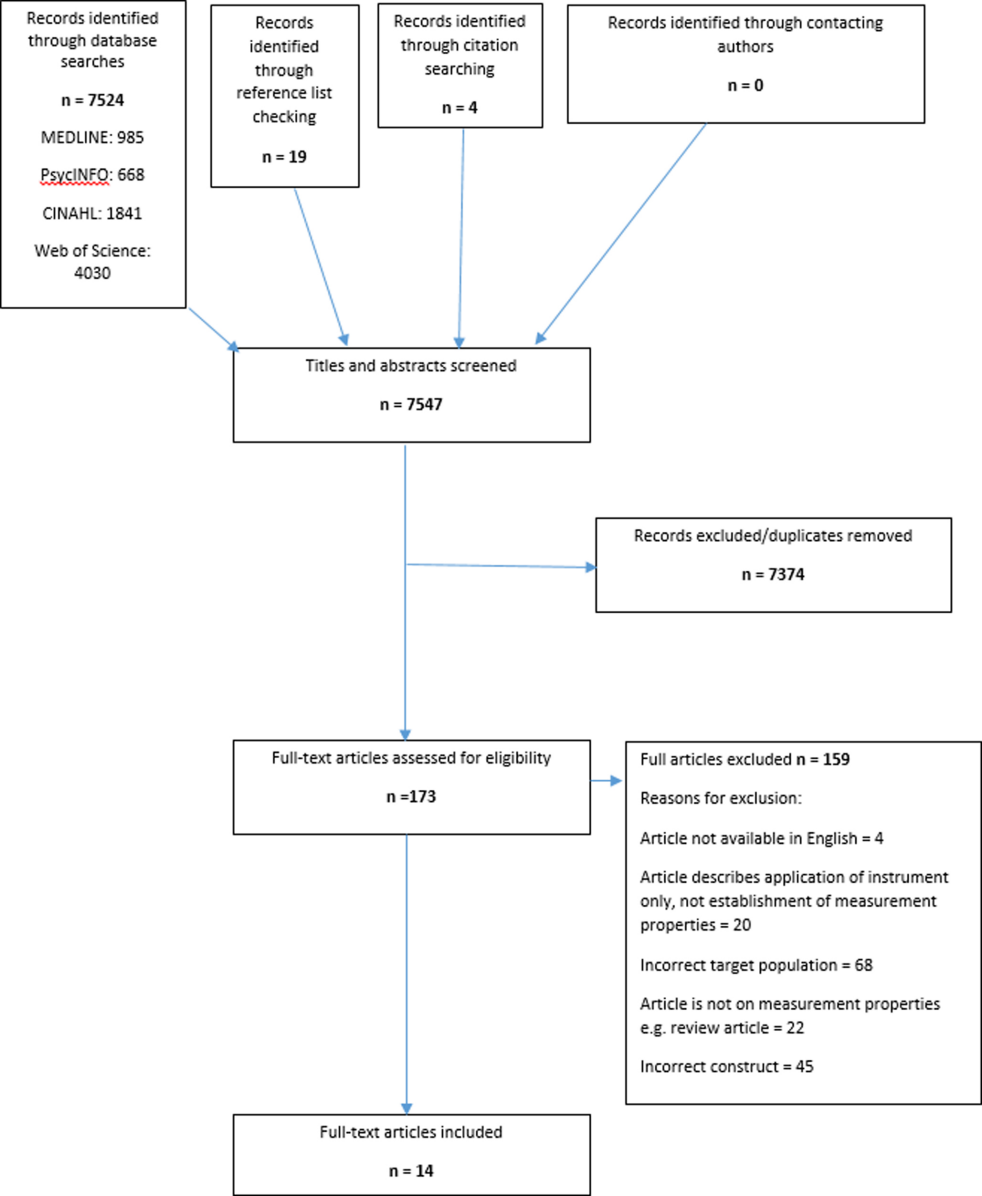
Results of literature search.

The literature search identified 7547 records for title and abstract screening (see Fig 1). Two researchers (JD and JR) independently screened the titles and abstracts against the inclusion criteria; 7374 were excluded (irrelevant or duplicates). If there was doubt regarding the eligibility of a study, the full paper was obtained for screening. One researcher (JD) screened full-text articles to assess eligibility: 159 articles were excluded. A list of excluded articles, with the reasons for exclusion, was maintained on bibliographic software and is available as a supplementary file. Fourteen articles, relating to 10 instruments were suitable for inclusion. The search process is documented in a flow chart, as recommended by the PRISMA statement[34] and COSMIN method [28] (Fig 1).

### Included instruments

The included instruments were compared in terms of their characteristics, the characteristics of included study populations, and the methodological quality and results of studies on their measurement properties. Table 3 and Table 4 summarise data extracted from included studies and relevant characteristics of study populations for studies with good or excellent methodological quality. Data for all studies included in the review are available in supplementary files (S4 and S5 Files).

#### 1. ASCOT-Carer: INT4 version

The included study [35] evaluated the acceptability, internal consistency, structural validity and construct validity of this instrument. Acceptability was judged to be satisfactory as the rate of missing values was <1%. Confirmatory factor analysis established that the seven domains captured the single underlying factor of social-care related quality of life. Cronbach’s α was 0.87, showing good internal consistency (defined as between 0.70 and 0.90[28]). Construct validity was assessed by testing hypotheses that the instrument’s scores would be positively associated with HRQoL and QoL, and negatively associated with carer strain. Statistically significant results in the expected directions were demonstrated: the weakest association was between SCRQoL and HRQoL, which authors felt was due to the absence of health-related domains in the instrument. The methodological quality of this study was fair for internal consistency and hypotheses testing (see Table 3).

This study has demonstrated that SCRQoL is a different construct to HRQoL, and therefore does not directly measure HRQoL or QoL. Limitations include the fact that carers of people with dementia comprised only 10% of the study population, and that >50% of carers in this study were under 65 years old (see Table 4). The authors recommend ASCOT-Carer:INT4 version for monitoring social care interventions and policy.

#### 2. Caregiver-targeted quality-of-life measure (CGQOL)

The included study [26] assessed the internal consistency, test-retest reliability, structural validity and construct validity of the CGQOL. The instrument was tested in both English and Spanish; cross-cultural validity was not evaluated and 71% of the sample were white [26]. The instrument’s acceptability was judged to be good. Assessment of structural validity using factor analysis led to the rejection of 11 items and subdivision of the domain for caregiving assistance. Floor and ceiling effects (using COSMIN’s 15% threshold[28]) were seen: ceiling effects on scores on the domains assistance with activities of daily living (27%) and spirituality and faith (26%); a floor effect was seen on the domain assistance with instrumental activities of daily living (20%). Cronbach’s α for each domain was between 0.78 and 0.94, indicating good internal consistency. Test-retest reliability was adequate for only 6 of the 10 domains. Methodological quality of the study was excellent for hypotheses testing and fair for other measurement properties evaluated (see Table 3). The instrument may prove burdensome to administer; the median time for administration in the included study was 23.5 minutes.

The authors concluded that further assessment of construct validity, reliability and responsiveness would be beneficial; this has not yet been undertaken (correspondence with author 19/6/16).

#### 3. Carers of older people in Europe (COPE) index

The study included in this review evaluates the acceptability, internal validity and construct validity of this instrument[36]. Construct validity was assessed by calculating correlations between the instrument’s subscales and other measures of health and wellbeing including the General Health Questionnaire (GHQ), a burden interview and a measure of caregiver competence and personal gain. The negative impact subscale correlated with depression, anxiety and strain and the positive value scale correlated with personal strain and gain in the expected directions. More than half of the carers felt that the instrument made it easier to discuss their needs and think clearly about the support they needed. All clinicians felt that use of the instrument made carers feel that their needs were of interest, and 70% felt that it improved their understanding of carers’ needs. The methodological quality of this study was fair for internal consistency and hypotheses testing (see Table 3).

Two other studies evaluating the psychometric properties of the COPE index in informal carers in several European countries were identified but did not meet our inclusion criteria [37, 38]. In both studies, internal consistency and criterion validity was better for the negative impact than for the positive impact scale, and Cronbach’s α was higher (>0.80) for the negative impact scale than in the study included in this review.

Further assessment of the validity of this instrument, particularly the positive impact scale, and establishment of its reliability and interpretability would be desirable.

#### 4. Caregiver quality of life instrument (CQLI)

One study reporting on the development of this instrument, and evaluation of its feasibility, test-retest reliability, construct validity and responsiveness was included [39]. A third of subjects reported it required considerable thought, which may suggest that it is cognitively demanding. Test-retest reliability was evaluated by calculating intra-class correlation coefficients for 28 carers, who were re-tested 2–3 weeks after initially completing the instrument: no significant differences were seen. Construct validity was assessed by testing hypotheses regarding expected differences in scores of the instrument between different states of wellbeing, different groups of carers (carers of cognitively impaired, physically impaired and well elderly relatives) and a measure of life stress. The instrument performed as expected. Responsiveness was assessed in 9 carers when the care recipient was admitted for respite care: the mean scores were significantly different in hypothesis testing, which the authors felt represented true change in the carer’s wellbeing. Methodological quality of this study was fair for reliability and poor for hypothesis testing and responsiveness (see Table 3).

More evidence on all measurement properties of this instrument would be required before recommending it for use by health and social care professionals.

#### 5. Carer Well-being and support questionnaire (CWS)

One study reporting on the development of this instrument and its psychometric evaluation in preliminary and final field testing was included in this review [40]. The first stage of assessment involved psychometric analysis of a pre-existing instrument (the Carers’ and Users’ expectation of services- carers’ version (CUES-C) and workshops with carers, and resulted in major changes to the instrument including refinement of its subscales and changing its name to more adequately reflect these. In the field tests, factor analysis and item reduction led to modification of CWS-v1 to CWS-v2, on which evaluations of acceptability, test-retest reliability and construct validity were made. A low percentage of missing data (<2% for each scale) was felt to reflect adequate acceptability. No floor or ceiling effects were observed in scores. Cronbach’s α was 0.96 and 0.97 for the wellbeing and support scales respectively, indicating that there may be a redundancy of items. Test-retest reliability was evaluated at 2 weeks in a sub-set of 92 carers, and showed satisfactory intra-class correlations for both scales. Construct validity was assessed by testing hypotheses regarding differences between scores on each scale and other measures of wellbeing and satisfaction with support; the scales performed as expected. The methodological quality of this study was excellent for all measurement properties except reliability (see Table 3).

Study limitations include a low response rate (36%) and lack of ethnic diversity in the sample (>90% were white) [40]. Further information on all measurement properties of this instrument including evaluation of its responsiveness would be desirable. The format of this instrument offers practical appeal as it is presented in booklet format with a section for undertaking a carer’s needs assessment.

#### 6. Impact of Alzheimer’s disease on caregiver questionnaire (IADCQ)

One study was identified reporting on evaluation of the internal consistency, reliability and structural validity of this instrument [41]. Assessment of structural validity using confirmatory factor analysis confirmed unidimensionality of the scale. Floor effects were seen for items regarding physical health, loneliness, worry, relationship with care recipient, relationship with family and friends, personal finances and sleep. Ceiling effects were seen for items regarding worry, frustration, social activities and stress. Cronbach’s α was satisfactory for all 12 items (0.917–0.928). Test-retest reliability at 4 weeks was evaluated in a subset of 50 carers; intra-class coefficients indicated moderate agreement. Construct validity was assessed by testing hypotheses regarding the relationship of scores on the IADCQ and scores on another instrument measuring HRQoL (SF-12v2): a moderate correlation in the expected direction was observed. Methodological quality of this study was excellent for internal consistency, good for reliability and fair for hypotheses testing (see Table 3).

Several issues would currently affect the suitability of this instrument for measuring HRQoL in dementia caregivers at present. Firstly, the construct does not measure positive aspects of caring (see Table 3). Secondly, the study population is younger than most others included in this review (see Table 4), and may therefore not be truly representative. The sample also consists only of carers of people with Alzheimer’s disease. Elimination of the floor and ceiling effects seen during this evaluation, by adding items to the upper and lower ends of the affected scales to discriminate between respondents, and assessment of construct validity in a larger sample of carers of people with all types of dementia would be advisable before recommending this instrument for use.

#### 7. Quality of life in Alzheimer’s disease (QOL-AD) questionnaire: Quality of life of the caregiver version (CQOL)

The QOL-AD was developed to assess quality of life in people with Alzheimer’s disease by combining the results of self-assessment by the person with dementia with proxy assessment by their primary carer [42]; there is also a scale for carers to self-assess quality of life (the CQOL). Measurement properties of the CQOL have not been reported on, although the authors state that validity has been established through comparison with depression and burden in the caregiver and other components of the QOL-AD[43]. Two studies were eligible for inclusion in this review reporting on evaluation of the measurement properties of the CQOL in informal carers of people with Alzheimer’s disease in Brazil [43, 44](see Table 4).

In the first study cross-cultural validity (between English and Portuguese versions of the instrument), internal consistency and test-retest reliability of the QOL-AD (including CQOL) were evaluated [44]. Cross-cultural equivalence of the instrument was not proven, and modifications to some of the items were made as a result. Satisfactory evidence of agreement was demonstrated for intra-examiner reliability (both statistics >0.90). Evidence for inter-examiner reliability was more modest (Pearson 0.93, Kappa 0.658). Cronbach’s α was 0.84 for the CQOL, providing satisfactory evidence for internal consistency. The methodological quality of this study was poor for internal consistency and reliability and fair for cross-cultural validity (see Table 3).

In the second study, internal consistency and construct validity of the Portuguese version of the instrument was assessed [43]. Construct validity was evaluated by comparing scores on other instruments measuring cognitive impairment of the care recipient (Mini Mental State Examination–MMSE), mental wellbeing of the both the caregiver and care recipient and caregiver burden (Neuropsychiatric Inventory, Geriatric Depression Score, Beck Depression Inventory, WHOQOL, Cornell Scale for Depression in dementia). Hypotheses regarding direction and size of effects were vaguely reported. Statistically significant relationships were demonstrated between CQOL score and all measures of mental wellbeing, but not between CQOL score and measurements of the patient’s quality of life, depression or cognitive impairment. The methodological quality of this study was fair for internal consistency and poor for hypotheses testing (see Table 3).

At present there is not sufficient evidence to recommend the use of the QOL-AD, CQOL version, for the assessment of Qol or HRQoL in informal carers of people with dementia. Further evaluation of the CQOL’s measurement properties in a representative sample of informal carers of people with all types of dementia would be desirable.

#### 8. Major mediating and outcome variables in caring questionnaire

One study evaluating the internal consistency and structural validity of this instrument was identified [45]. Cronbach’s α was within a satisfactory range (0.70 to 0.90) for 16 of the 19 scales. Exploratory factor analysis found that multiple factors existed within each domain. The methodological quality of this study was fair for internal consistency and structural validity (see Table 3).

This instrument is not suitable for use in practice but may be useful in research. The structure of this instrument is burdensome (see Table 3). Construct is also an issue: as this instrument is a compilation of many others, it may measure a collection of constructs relevant to caregiving rather than QoL or HRQoL. Finally, generalisability of the results is limited by the fact that the proportion of dementia caregivers in this sample was unknown, and the age ranges of both care recipients and carers in this study was much wider than in others included in this review.

#### 9. Medical outcomes study short-form health survey (SF-36)

The SF-36 has proven validity and reliability [19], and is widely used. The study included in this review evaluates the internal consistency and structural validity of the Argentinian version of the instrument (see Table 4) though factor analysis and hypothesis testing [31]. Cronbach’s α was acceptable for all scales (0.72–0.92). Hypotheses tests compared SF-36 scores to scores on instruments measuring caregiver burden (Zarit Burden Interview), depression (Neuropsychiatric Inventory) and cognitive impairment of the care recipient (MMSE and Clinical Dementia Rating). Statistically significant correlations in the expected directions were observed. The methodological quality of this study was good for internal consistency and structural validity and poor for hypotheses testing (see Table 3).

At present, there is insufficient evidence on the measurement properties of the SF-36 to recommend it for use in informal carers of people with dementia. The findings of the study included in this review are not generalizable, as the Argentinian version of the instrument was used and only carers of people with Alzheimer’s disease were included. Further evaluation of this instrument in a larger population of informal carers of people with all types of dementia including assessment of its responsiveness would be desirable.

#### 10. Caregiver well-being scale

Four studies on the measurement properties of this instrument are included in this review, reporting on: instrument development [46] and evaluation of its internal consistency [46, 47], structural validity [46, 47], and attempts to shorten the instrument [48, 49]. In first study [46] (see Table 3 and Table 4), Cronbach’s α for the instrument overall, and for each subscale was 0.94 indicating good internal consistency. Construct validity was investigated by testing hypotheses comparing the instrument’s scores to those of a life satisfaction questionnaire: statistically significant associations in the expected directions were seen. Hypotheses were also tested regarding how scores would differ between caregivers and non-caregivers. As expected, caregivers were found to meet their basic needs significantly less than non-caregivers for all items except those on attendance to physical needs. Although differences were seen between the groups in the expected direction on the activities of daily living scale, these did not reach statistical significance. Methodological quality of this study was excellent for internal consistency and structural validity, and fair for hypotheses testing (see Table 3).

Using the same study population (see Table 4), the second included study [47] investigated the internal consistency and structural validity of the instrument using structural equation modelling. The methodological quality of this study was excellent for both measurement properties (see Table 3).

The final 2 studies included in this review report on attempts to shorten the instrument to 18 items to make it less burdensome (see Table 3 and Table 4). In one study, the content validity of the instrument was assessed by an expert panel [48]. Both scales were shown to have good reliability: inter-rater agreement was 89% for the needs dimension and 100% for the activities dimension. Strong content validity and factorial validity indices for each scale were also reported. The other study assesses internal consistency and structural validity of the shortened instrument [49]. Cronbach’s α was 0.83 overall for the shortened instrument. Evidence on the instrument’s reliability, cross-cultural validity and responsiveness is lacking. The methodological quality of these studies was excellent (see Table 3).

### “Best evidence” synthesis of measurement properties

Using the “levels of evidence” approach [28], a table combining the results of studies on the measurement properties of the included instruments and assessment of their methodological quality has been produced (see Table 5). This shows the CWS [40] as the instrument with evidence of the highest quality for the greatest number of measurement properties, followed by the Caregiver Well-being Scale [46–49]. However, this approach does not account for the numbers of studies of excellent methodological quality: for the Caregiver Well-being scale 4 studies examined 4 measurement properties; for the CWS 5 measurement properties were examined in 1 study.

**Table 5:** "Levels of evidence" for measurement properties of included instruments.

Instrument	Internal consistency	Reliability	Measurement error	Content validity	Structural validity	Hypotheses testing	Cross-cultural validity	Criterion validity	Responsiveness
ASCOT-Carer INT4	+	-	-	-	+	+	-	-	-
CGQOL	+	+	-	-	+	+++	-	-	-
COPE index	+	-	-	-	-	+	-	-	-
CQLI	-	+	-	-	-	?	-	-	?
CWS	+++	++	-	+++	+++	+++	-	-	-
IADCQ	+++	++	-	-	+++	+	-	-	-
QOL-AD: CQOL	+	?	-	-	-	poor	+	-	-
Major mediating and outcome variables in caring questionnaire	+	-	-	-	+	-	-	-	-
SF-36	++	-	-	-	++	+	-	-	-
Caregiver Well-being scale	+++	-	-	+++	+++	+	-	-	-

+ = limited positive rating.

? = only studies of poor methodological quality.

++ = moderate positive rating.

- = not evaluated.

+++ = strong positive rating.

## Discussion

This systematic review reveals that currently the CWS is the most appropriate instrument to measure quality of life in informal carers of people with dementia; however further evaluation of its reliability, cross-cultural validity and responsiveness would be desirable. Its format may also be advantageous for health and social care professionals, as it includes a needs assessment component. The Caregiver Well-Being Scale is also worthy of consideration. Although there is evidence for only 4 of its measurement properties, these have been explored in a greater number of studies than the CWS, and in larger, although more heterogeneous, populations of carers. Authors have reported receiving over 1000 requests for use of this instrument in clinical and research activities [49].

Both the CWS and the Caregiver Well-Being Scale are descriptive instruments, and therefore may be most useful to researchers and health and social care practitioners. If an index measurement for use in economic evaluations was required, the IADCQ could be considered. Compared to the other index measures included in this review, the IADCQ has the best quality evidence for the greatest number of measurement properties. However, the only study on this instrument includes carers of people with Alzheimer’s disease only: evaluation of its measurement properties in a population of informal carers of people with all types of dementia would be necessary before recommending it. This review found limited data on generic instruments in common use in informal carers of people of dementia: only 2 studies on measurement properties of the SF-36 were included.

Considering the evidence for the measurement properties of the included instruments, it is striking that there is a lack of evidence for many properties considered relevant in COSMIN methodology. The only property for which all instruments had some evidence was internal consistency. Only 5 instruments had evidence for reliability, 2 had evidence for content validity, and 1 had evidence for cross-cultural validity and responsiveness. This is consistent with findings in other systematic reviews [14, 15, 25]. Authors of these reviews concluded that more thorough testing of instruments for people with dementia and their carers was necessary; the findings of this review support this [15].

## Limitations

The findings of this review are limited by the often poor methodological quality of included studies. Poor reporting of the handling of missing data was noted in 6 of the 14 included studies [26, 35, 36, 39, 44, 45]. Small sample size affected quality assessment in 7 included studies [26, 31, 36, 39, 40, 43, 44]. In 4 studies there was insufficient evidence on the measurement properties of the comparator instrument used [35, 36, 39, 43]. Vague specification of hypotheses a priori affected the quality of 4 studies [31, 36, 43, 46]. Variable quality of included studies and small sample size were also noted in a systematic review of quality of life instruments for people with dementia [15]. There is also considerable variation in the settings and samples of included studies. Four studies [31, 41, 43, 44] included only carers of people with Alzheimer’s disease, meaning that the results would not be generalizable to informal carers of people with other types of dementia. The studies on the QOL-AD (CQOL version) [43, 44] and the SF-36 [31] used versions of the questionnaires in Portuguese and Argentinian respectively. It would therefore not be possible to generalise these results to an English-speaking population. Ethnicity was only reported in 2 studies; this is important as there is evidence that carers in ethnic minority groups may prioritise dimensions of an instrument differently [26].

Other limitations of this review include the decision to include only studies published in English, and the fact that only one reviewer (JD) extracted data from included studies. These decisions were made due to restriction of time and resource, attributable to this project being a dissertation for a Masters degree. Pilot data extraction of one included study was undertaken alongside a supervisor with expertise with COSMIN methodology (EM); nonetheless the authors recognise that this limits the robustness of our findings.

## Conclusion and recommendations

The CWS is the most appropriate instrument to use to measure quality of life in informal carers of people with dementia at present. This measure is descriptive, and may be of most use to health and social care professionals. There is not currently an index measure which could be recommended for use in economic analyses; the IADCQ and ASCOT-Carer INT4 are promising but require further evaluation in informal carers of people with dementia in the UK. All instruments included in this review would benefit from more rigorous evaluation of their measurement properties, in ethnically representative samples of carers of people with all types of dementia. Improved reporting of quality assessment criteria, and wider adherence to a rigorous rating scheme such as COSMIN would significantly improve the quality of the evidence on this subject and improve the robustness of recommendations made, and assist all stakeholders in choosing the most appropriate instrument for their purpose.

## Supporting information

S1 File Project protocol. (DOCX)

S2 File Search strategies. (DOCX)

S3 File Excluded articles. (DOCX)

S4 File Characteristics of included instruments and summary of quality assessment. (DOCX)

S5 File Characteristics of included studies. (DOCX)

S6 File Prisma 2009 checklist. (DOC)
