# Supplementary material for: How best to assess quality of life in informal carers of people with dementia; A systematic review of existing outcome measures
Source: PLoS One. 2018 Mar 14;13(3):e0193398. doi: 10.1371/journal.pone.0193398 (PMC5851581; doi:10.1371/journal.pone.0193398)
Supplement: S1 File — (DOCX) [file pone.0193398.s001.docx]

**Measurement of psychological wellbeing and quality of life in informal carers of people with dementia: a systematic review of current instruments**

**1. Introduction**

Dementia is a syndrome in which there is chronic and progressive disturbance of multiple higher cortical functions of the brain, causing impairment in memory, thinking, orientation, comprehension, calculation, learning capacity, language and judgement(1). These can be accompanied by deterioration in emotional control, social behaviour and motivation(1). The most common cause of dementia is Alzheimer’s disease, which accounts for 62% of cases(2). Other causes include vascular dementia, lewy-body dementia, frontotemporal dementia and Parkinson’s dementia. Mixed forms of dementia account for 10% of cases(2), and boundaries between subtypes can be unclear(1).

Worldwide, the total number of people with dementia was estimated to be 35.6 million in 2010(1). The prevalence of dementia is expected to double every 20 years, with 65.7 million cases expected by 2030 and 115.4 million by 2050(1). Dementia is associated with increasing age, its prevalence doubling with every 5 year increment after the age of 65, and is the leading cause of dependency and disability in older adults across the world(1). Dementia has therefore been identified by the World Health Organisation as a public health priority(1). Many countries, including the UK, are recognising the global health challenge presented by dementia, and implementing policies, plans or legislation which aim to improve the health and social care of people with dementia and their carers(1). Dementia also presents an economic challenge: the estimated cost to the UK at present is £26 billion per year(2). Eleven billion pounds of this figure (44 %) represents the unpaid work of carers of people with dementia(2). Carers are therefore a vital resource to the economy as well as the person they care for, and will become even more so as the prevalence of dementia increases in the ageing population.

In the UK, there are currently 800 000 people living with dementia(2). Two thirds of people live in the community, supported by 670 000 informal (unpaid) carers. The Royal College of General Practitioners (RCGP) define a carer as a person *“who provides unpaid support to a partner, child, relative or friend who couldn’t live independently without that support or whose health or wellbeing would deteriorate without this help. This could be due to frailty, disability, or serious health condition, mental ill health or substance misuse”(3)*.

Caring for a person with dementia is an experience which is likely to begin insidiously, and evolve and change as the disease progresses. Each individual with dementia will have different care needs, depending on factors such as the type of dementia, the presence of behavioural, psychological or other physical conditions, and also their personality, relationships and habits prior to having dementia(1). Generally however, caring in the early stages may involve prompting, reminding and assisting the person with dementia to maintain their independence(1). Later it may involve help with activities such as dressing appropriately and food preparation. In the later stages, caring for a person with dementia involves the provision of care, support and supervision 24 hours a day and providing full assistance with activities such as eating, drinking, bathing and toileting(1). At all stages, caring may involve the management of behavioural and psychological symptoms.

The experience of being a carer has been viewed and measured in many different ways. It is important to recognise that most carers develop and accept their role within the context of a longstanding relationship which predates the onset of the condition(s) for which care recipient requires care(4). In a survey of nearly 6 000 carers in 6 European countries in 2006, 57% identified love and affection as their motivation for caring, with only 3% stating they had no alternative(5). In some caregiving literature, caregiving has therefore be conceptualised in a positive way, and described in terms of “positive gains.” These include improved self-esteem and satisfaction from the process of providing care(6). Increased closeness and mutual affection with the care recipient have also been described (4, 7).

Caring can also involve a significant of expenditure of time and energy over prolonged periods of time, dealing with tasks which can be physically and mentally challenging(1). Negative effects of caring on psychological, physical, social and economic wellbeing are described consistently in caregiving literature(8, 9), and taken into consideration in health care policy and guidelines for healthcare practitioners and policy makers. These are summarised in Table 1. Several factors have been identified that make carers more vulnerable to negative effects, including behavioural problems and depression of the care recipient, cohabiting with the care recipient and social isolation(10). Carers of people with dementia often deal with many of these factors simultaneously and thus represent a vulnerable group of carers(10).

Table 1: Negative effects of caring

| **Physical**  Poorer self-rated health, greater medication use(1, 8)  Obesity, higher metabolic risk, higher levels of stress hormones, compromised immune response(1) | **Psychological**  Burden, distress, anxiety, depression(10) |
| --- | --- |
| **Social**  Social stigma, abandonment of hobbies(10) | **Economic**  Costs of professional care,  Loss of earnings of carer and person with dementia(10) |

The effects of being a carer have also been explored in research into their impact on quality of life. Quality of life is a concept which reflects a person’s physical and psychological health, personal beliefs and relationships(11), and allows us to evaluate our lives in a way which encompasses all of these factors(12). A recent survey of 195 364 informal carers in England found that they reported poorer health-related quality of life than non-carers of similar age, gender, ethnicity and social strata, and that lower quality of life was related to higher time commitment of the carer to their role(13). In studies of carers of people with dementia, factors such as living with the person with dementia(11, 14) and depressive illness in the person with dementia(14) have been shown be associated with poorer quality of life.

There are many recommendations in the literature and in clinical guidance about how healthcare practitioners can support carers of people with dementia. The WHO recommends that carers should be comprehensively assessed to allow the selection of interventions suitable for the carer, that these should be implemented flexibly, and their outcomes measured frequently to allow for adjustment for the individual(1). At national level, there are indicators in the adult and social care outcomes framework and the quality and outcomes framework, with more in development, to encourage local councils and health practitioners to improve their integration and address the impact of caregiving on the carer(15-17). The RCGP has produced a package advising general practices of how they can organise their practice to support carers(3). In the literature, it is recommended that general practitioners (GPs) should use instruments to identify carers at risk of anxiety and depression in order to provide support(13), consider factors specific to the caregiving situation which may affect the consequences to the carer(4), and prescribe psychosocial interventions as specifically as they prescribe medication(8).

The purpose of this systematic review is to identify instruments which assess quality of life in carers of people with dementia and assess their measurement properties, in order to suggest which instruments would be the best to use in practice to aid assessments, base recommendations and monitor the outcomes of interventions offered.

**Aims and Objectives**

i) To identify instruments in current use which measure quality of life in informal carers of people with dementia

ii) To assess the psychometric properties of instruments identified, with a view to establishing the best instrument(s) to use in practice.

**2. Methods**

**2.1 Study characteristics**

**2.1.1 Population**

Informal carers of people with dementia.

**2.2.2 Intervention**

Health-related self-report instruments used to measure quality of life in informal in carers of people with dementia. Health-related self-report instruments measure aspects of health as directly assessed by the patient, without interpretation by any other individual, and may be administered by interview, computer or by the patient themselves. Quality of life will be defined as *“an evaluation of all aspects of [life] encompassing life factors such as family circumstances, financing and job satisfaction(18).”* Instruments which examine health-related quality of life, defined as *“aspects of our lives that are dominated or significantly influenced by our mental or physical wellbeing(18)”* will also be included.

Inclusion criteria:

i) Instruments in which status is reported directly by the carer without interpretation by any other individual.

ii) Instruments based on a theoretical framework which have been created to assess quality of life in informal carers of people with dementia.

iii) Instruments which have been developed and validated using a population of carers of people with dementia, or instruments developed and validated in a different population which have subsequently had psychometric properties established in a population of carers of people with dementia.

Exclusion criteria:

i) Instruments which measure status of person with dementia only.

ii) Instruments which measure status of whole family or carer-care recipient dyad only.

iii) Instruments based on another instrument, or compiled of parts of other instruments, for which psychometric properties have not been established.

iv) Instruments which seek factual information only e.g. number of hours spent caring, tasks performed by carer, without appraisal of these by carer with reference to psychological wellbeing or quality of life.

vi) Instruments whose psychometric properties have not been tested in a population of carers of people with dementia.

**2.2.3 Outcomes**

These will be the psychometric outcomes available for the instruments identified and will relate reliability, validity and responsiveness.

**2.2.5. Studies**

Inclusion criteria:

i) Studies reporting the development and/or measurement properties of an instrument. Articles on instrument development will be included, as they may contain information about the construct measured and supply information which is needed to evaluate the instrument’s content validity(19).

Exclusion criteria:

i) Studies published in a language other than English

ii) Studies which report only on the application or use of an instrument, without establishment of its psychometric properties.

iii) Studies in which the instrument of interest is used to validate other instruments, or used in an interventional study such as and RCT or longitudinal study. It can be difficult to find these studies, and to interpret the evidence for validity or responsiveness within them.(19)

**3. Search strategy**

**3.1 Electronic searches**

The following databases will be searched from the earliest possible date to the present day:

i) MEDLINE

ii) EMBASE

iii) LILACS

iv) PubMed

v) Science Citation Index

vi) Cochrane Diagnostic Test Accuracy Working Group

vii) Web of Science

viii) CINAHL

ix) PSYCinfo

x) Cochrane Dementia and Cognitive Improvement Group Register

xi) International Alzheimer’s Disease Research Portfolio (IADRP)

xii) ASSIA, CSA sociological abstracts, CSA social services abstracts

xiii) AgeInfo

xiv) Ageline

xv) SCOPUS

xvi) SCIE (Social Care Institute for Excellence)

xvii) TRIP

xviii) Abstracts in Social Gerontology

xix) Health and Psychosocial Instruments database

Grey literature including unpublished studies, theses and conference presentations will be identified by searching

i) Open Grey (System for Information on Grey Literature in Europe)

ii) Index to Theses

iii) Relevant websites including The Alzheimer’s Society, Alzheimer Research UK, Carers Direct, Carers UK, Carers Trust and Dementia UK.

**3.2 Electronic search strategy**

A structured search strategy will be developed using indexing terms and free text. Indexing terms for patient reported outcomes differ between databases, and multiple search terms are likely to be necessary(12). Search strategies will concentrate on terms for the constructs of interest (psychological wellbeing and quality of life), carers of people with dementia and measurement properties(19). A list of synonyms will be compiled for each of these and combined with them using the OR synonym, and these searches combined using the AND synonym(19). To identify studies that focus only on measurement properties, a filter may be used for searching Medline and PubMed.(19) Reference lists of identified studies will be checked manually. If many new studies are identified in this way the electronic search strategy will be improved and repeated(19). An information expert will be consulted for advice on the search strategy. A full electronic search strategy for at least one database will be presented such that the search can be repeated and the comprehensiveness of the search strategy assessed(19).

**3.3 Searching other sources**

Reference lists from primary studies and review articles identified from electronic searches will be searched. Key journals identified by electronic searching of databases will be hand searched for relevant articles. Citation searching of key papers will also be undertaken(20).

**4. Study selection**

Inclusion criteria:

i) Participants: informal carers of people with dementia ii) Intervention: instruments used to measure quality of life in informal carers of people with dementia

ii) Outcomes: psychometric properties of instruments identified

iii) Studies: primary studies of informal carers of people with dementia.

Exclusion criteria:

i) Studies not published in the English language.

Inclusion and exclusion criteria will be piloted and refined to ensure they are applied consistently(20).

**4.1 Stage 1: Title and abstract screening**

Titles and abstracts will be read independently by 2 researchers and assessed against the inclusion criteria.

**4.2 Stage 2: Full paper screening**

Full papers will be obtained for detailed assessment against the inclusion criteria if stage 1 suggests they meet the criteria, or if this decision cannot be made at stage 1. Duplicate studies will be removed. Where multiple reports of the same study are identified, these will be treated as a single study and reference made to all publications(20). All papers will be independently assessed by 2 researchers to improve reliability of the decision making process(20). If there is insufficient information to decide whether a study meets the inclusion criteria, authors will be contacted for further information. If authors fail to respond by a certain date, the study will be excluded and listed as a “potentially relevant study.”(20) A formal assessment of agreement between researchers will be undertaken using a Kappa statistic(20). Disagreements regarding study eligibility will be discussed and resolved by consensus if possible, a third researcher will be consulted if needed.

A list of articles excluded at stage 1 and 2 will be maintained on bibliographic software (Endnote) and reasons for exclusion documented.

**5. Data extraction**

Studies identified for inclusion will be examined independently by 2 researchers using a data extraction form, which will be piloted prior to the study(20). Disagreements between researchers will be resolved by discussion, a third researcher will be involved if necessary(20). Authors will be contacted for further information on studies if needed(20).

**6. Quality assessment**

To assess the quality of included studies, the Consensus-based Standards for the selection of health status measurement instruments (COSMIN) checklist(21) will be used. This consensus-based checklist can be used evaluate the methodological quality of studies on health measurement instruments including health-related patient reported outcomes(21). It can be used in systematic reviews which assess the quality of studies on measurement properties of instruments with a similar purpose, enabling comparison between studies and selection of the best instrument(22). Checklist items relate to 3 domains: reliability, validity and responsiveness(21). The checklist contains certain items which require subjective judgement e.g. appropriate time intervals, adequate description of reference instruments (21, 23). These items will be discussed with the project supervisor(s), and agreement reached on how to handle them before the checklist is used for the review.

**7. Data Synthesis**

**7.1 Results of literature searches**

The literature search will be described in full. The number of references identified and number of studies considered eligible for inclusion after abstract and full paper screening will be stated, and the process illustrated in a flow diagram as per the Preferred Reporting Items for Systematic Reviews and Meta-analyses (PRISMA) statement(24). Reasons for exclusion of studies will be given, and a list of excluded studies added to the project as an appendix.

**7.2 Results of quality assessment**

A summary table will be presented to give an overview of the quality of included studies(25). A narrative summary will discuss included studies in terms of their general quality, variability of quality between studies and important weaknesses identified in individual studies(12, 25). The rating of each study against the criterion used for quality assessment will be presented in an appendix to the project.

**7.3 Results from included studies**

Study characteristics, including study quality, population, setting, the language and version of the instrument used and method of administration will be assessed in order to establish whether there is homogeneity between studies which would allow quantitative pooling of study results.(19)

Narrative synthesis will be used to describe the results of included studies, explore relationships within and between studies, evaluate the robustness of the synthesis(20), and to synthesise results on properties for which quantitative data is unavailable e.g. content validity(19). Characteristics of the measurement instruments identified and included study populations will be summarised in a table(19).

Each instrument identified will be examined in turn. Tables will be constructed showing all psychometric properties of each instrument identified(19). It is hoped that recommendations can be made regarding which instrument(s) should be used in practice, taking account of both the measurement properties of the instrument and the methodological quality of included studies.(19)

**8. Ethical Considerations**

Ethical approval will not be required in order to undertake this systematic review. Included studies will be examined for information on how ethical approval for the study was obtained and how consent from participants was sought.

**9. Timetable**

**
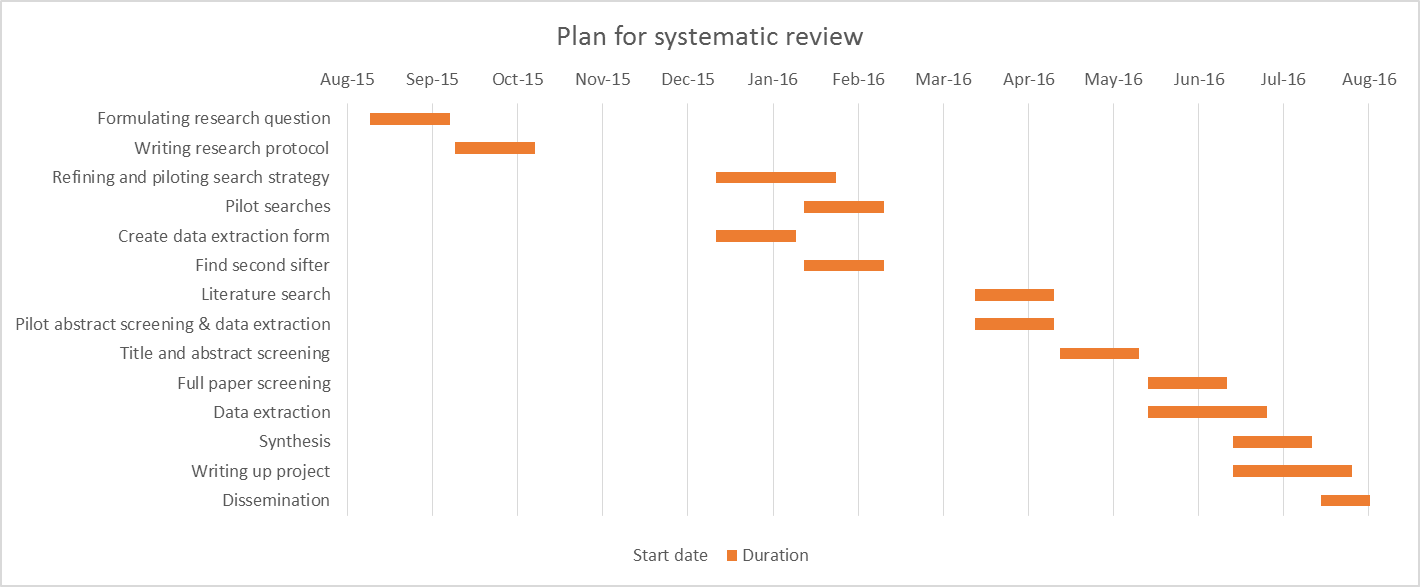
**

**10. Costs**

It is anticipated that costs may be incurred by requesting inter-library loans, photocopying and printing. The cost of some inter-library loans may be covered by Newcastle University, all other costs will be covered personally.

**11. Risks**

**11.1 Timely execution of the project**

A Gantt chart has been constructed to aid with the timely execution of the project. Following submission of the finalised protocol, this will be reviewed with the supervisors of the project and adjustments made if needed. Regular meetings with supervisors will be arranged to review progress. It is anticipated that a second supervisor, second sifter and information expert may need to be identified to support this review: following finalisation of the protocol these individuals will be identified as soon as possible to facilitate timely running of the project.

**11.2 Literature Search**

Following pilot searching, the aims and objectives of the review will be reviewed and consideration given to the number of papers identified and the timeframe of the project. If it is necessary in order to complete the project on time, the search may be further limited, e.g. by date of publication. If this is necessary the limitations and implications of this will be discussed in both the methods section and the project management appendix of the dissertation (CRD). Advice will be sought from supervisors should this be required.

**11.3 Expertise**

In order to search comprehensively for relevant instruments, advice from an information expert will be sought.

**12. References**

1. World Health Organisation, Alzheimer's Disease International. Dementia: a Public Health Priority: World Health Organisation; 2012 [Available from: <http://apps.who.int/iris/bitstream/10665/75263/1/9789241564458_eng.pdf?ua=1>.

2. Alzheimer's Society. Dementia 2014 Infographic: Alzheimer's Society; 2014 [Available from: <http://www.alzheimers.org.uk/infographic>.

3. Royal College of General Practitioners, Princess Royal Trust. Supporting Carers: an action guide for general practitioners and their teams: Royal College of General Practitioners, The Princess Royal Trust for Carers; 2011 [Second:[Available from: <http://www.rcgp.org.uk/clinical-and-research/clinical-resources/carers-support.aspx>.

4. Ablitt A, Jones GV, Muers J. Living with dementia: A systematic review of the influence of relationship factors. Aging & Mental Health. 2009;13(4):497-511.

5. EUROFAMCARE. Services for Supporting Family Carers of Older Dependent People in Europe: Characteristics, Coverage and Usage. The Trans-European Survey Report. 2006.

6. Harkness KI, Tranmer JE. Measurement of the Caregiving Experience in Caregivers of Persons Living With Heart Failure: A Review of Current Instruments. Journal of Cardiac Failure. 2007;13(7):577-87.

7. Cheng S-T, Mak EPM, Lau RWL, Ng NSS, Lam LCW. Voices of Alzheimer Caregivers on Positive Aspects of Caregiving. The Gerontologist. 2015.

8. Brodaty H, Green A. Who cares for the carer? The often forgotten patient. Australian Family Physician. 2002;31(9):1-4.

9. Schulz R, Martire LM. Family Caregiving of Persons With Dementia: Prevalence, Health Effects, and Support Strategies. The American Journal of Geriatric Psychiatry. 2004;12(3):240-9.

10. Brodaty H, Hadzi-pavlovic D. Psychosocial Effects on Carers of Living with Persons with Dementia. Australian and New Zealand Journal of Psychiatry. 1990;24(3):351-61.

11. Bruvik FK, Ulstein ID, Ranhoff AH, Engedal K. The quality of life of people with dementia and their family carers. Dement Geriatr Cogn Disord. 2012;34(1):7-14.

12. Higgins JP, Green S. Cochrane Handbook for Systematic Reviews of Interventions: The Cochrane Collaboration; 2011. Available from: <http://www.cochrane-handbook.org/>.

13. Thomas GPA, Saunders CL, Roland MO, Paddison CAM. Informal carers’ health-related quality of life and patient experience in primary care: evidence from 195,364 carers in England responding to a national survey. BMC Family Practice. 2015;16:62.

14. Orgeta V, Orrell M, Hounsome B, Woods B. Self and carer perspectives of quality of life in dementia using the QoL-AD. Int J Geriatr Psychiatry. 2015;30(1):97-104.

15. Department of Health. Adult social care outcomes framework (ASCOF) 2015 to 2016: Department of Health; 2014 [Available from: <https://www.gov.uk/government/publications/adult-social-care-outcomes-framework-ascof-2015-to-2016>.

16. NHS Employers. 2015/16 General Medical Services (GMS) contract Quality and Outcomes Framework (QOF): Guidance for GMS contract 2015/16: NHS Employers, British Medical Association, NHS England; 2015 [Available from: <http://www.nhsemployers.org/~/media/Employers/Documents/Primary%20care%20contracts/QOF/2015%20-%2016/2015%2016%20QOF%20guidance%20for%20stakeholders.pdf>.

17. Health and Social Care Information Centre. NHS Outcomes Framework Indicators - May 2015 release: Health and Social Care Information Centre; 2015 [Available from: <http://www.hscic.gov.uk/article/2021/Website-Search?productid=17975&q=nhs+outcomes+framework+indicators+2015&sort=Relevance&size=10&page=1&area=both#top>.

18. Patrick DL, Guyatt GH, Acquadro C. Chapter 17 Patient-reported outcomes. In: Higgins JP, Green S, editors. Cochrane Handbook for Systematic Reviews of Interventions Version 510: The Cochrane Collaboration; 2011.

19. de Vet HCW, Terwee CB, Mokkink LB, Knol DL. Reviews of measurement properties. Measurement in Medicine: a practical guide. 1. Cambridge: Cambridge University Press; 2011. p. 338.

20. Centre for Reviews and Dissemination. Systematic Reviews: CRDs Guidance for Undertaking Reviews in Health Care.: University of York; 2008 [Available from: <http://www.york.ac.uk/crd/guidance/>.

21. Mokkink LB, Terwee CB, Patrick DL, Alonso J, Stratford PW, Knol DL, et al. The COSMIN checklist for assessing the methodological quality of studies on measurement properties of health status measurement instruments: an international Delphi study. Quality of Life Research. 2010;19(4):539-49.

22. Mokkink L, Terwee C, Knol D, Stratford P, Alonso J, Patrick D, et al. The COSMIN checklist for evaluating the methodological quality of studies on measurement properties: A clarification of its content. BMC Medical Research Methodology. 2010;10(1):22.

23. Mokkink L, Terwee C, Gibbons E, Stratford P, Alonso J, Patrick D, et al. Inter-rater agreement and reliability of the COSMIN (COnsensus-based Standards for the selection of health status Measurement Instruments) Checklist. BMC Medical Research Methodology. 2010;10(1):82.

24. Moher D, Liberati A, Tetzlaff J, Altman DG, The PG. Preferred Reporting Items for Systematic Reviews and Meta-Analyses: The PRISMA Statement. PLoS Med. 2009;6(7):e1000097.

25. Reitsma J, Rutjes A, Whiting P, Vlassov V, Leeflang MMG, Deeks JJ. Assessing methodological quality. In: Deeks JJ, Bossuyt PM, Gatsonis C, editors. Cochrane Handbook for Systematic Reviews of Diagnostic Test Accuracy: The Cochrane Collaboration; 2009. p. 1-24.
