# Supplementary material for: How best to assess quality of life in informal carers of people with dementia; A systematic review of existing outcome measures
Source: PLoS One. 2018 Mar 14;13(3):e0193398. doi: 10.1371/journal.pone.0193398 (PMC5851581; doi:10.1371/journal.pone.0193398)
Supplement: S2 File — (DOCX) [file pone.0193398.s002.docx]

# Appendix 1: Search Strategy for MEDLINE

1. exp Dementia/

2. exp Dementia, Multi-Infarct/

3. exp Frontotemporal Dementia/

4. exp Cognition Disorders/

5. exp Memory Disorders/

6. Alzheimer*.mp.

7. Dementia*.mp.

8. cognitive disorder*.mp.

9. chronic mental disorder*.mp.

10. mental frail*.mp.

11. Huntington*.mp.

12. CJD*.mp.

13. Binswanger*.mp.

14. Korsakoff*.mp.

15. Wernicke*.mp.

16. Lewy*.mp.

17. Creutzfeld*.mp.

18. cognitive impairment.mp.

19. or/1-18

20. exp "Quality of Life"/

21. exp Personal Satisfaction/

22. quality of life.mp.

23. QoL.mp.

24. Health-related quality of life.mp.

25. life satisfaction.mp.

26. well being.mp.

27. wellbeing.mp.

28. HRQoL.mp.

29. 20 or 21 or 22 or 23 or 24 or 25 or 26 or 27 or 28

30. exp Caregivers/

31. exp Family/

32. exp Friends/

33. Interpersonal Relations/

34. exp "Child of Impaired Parents"/

35. carer*.mp.

36. care giver*.mp.

37. caregiv*.mp.

38. informal care*.mp.

39. parent*.mp.

40. mother*.mp.

41. father*.mp.

42. son*.mp.

43. daughter*.mp.

44. grandparent*.mp.

45. husband*.mp.

46. wife.mp.

47. Spouse*.mp.

48. family.mp.

49. families.mp.

50. friend*.mp.

51. neighbour*.mp.

52. relative*.mp.

53. or/30-52

54. 19 and 29 and 53

55. (Validation Studies or Comparative Study).pt.

56. Psychometrics/is, mt or "Outcome Assessment (Health Care)"/mt or "Health Status Indicators"/ or "reproducibility of results"/ or "discriminant analysis"/

57. psychometr*.ti,ab. or clinimetr*.tw. or clinometr*.tw.

58. (cronbach* and (alpha or alphas)).ti,ab.

59. (item and (correlation* or selection* or reduction*)).ti,ab.

60. (item and (correlation* or selection* or reduction*)).ti,ab.

61. precise values.ti,ab.

62. test-retest.ti,ab.

63. (test and retest).ti,ab.

64. (reliab* and (test or retest)).ti,ab.

65. (stability or interrater or inter-rater or intrarater or intra-rater or intertester or inter-tester or intratester or intra-tester or interobserver or inter-observer or intraobserver or intra-observer or intertechnician or inter-technician or intratechnician or intra-technician or interexaminer or inter-examiner or intraexaminer).ti,ab.

66. (intra-examiner or interassay or inter-assay or intraassay or intra-assay or interindividual or inter-individual or intraindividual or intra-individual or interparticipant or inter-participant or intraparticipant or intra-participant or kappa or kappa?s or kappas or repeatab*).ti,ab.

67. ((replicab* or repeated) and (measure or measures or findings or result or results or test or tests)).ti,ab.

68. (generaliza* or generalisa* or concordance).ti,ab.

69. (intraclass and correlation*).ti,ab.

70. known group.ti,ab.

71. factor analysis.ti,ab.

72. factor analyses.ti,ab.

73. (dimension* or subscale* or discriminative).ti,ab.

74. (multitrait and scaling and (analysis or analyses)).ti,ab.

75. item discriminant.ti,ab.

76. interscale correlation*.ti,ab.

77. (error or errors).ti,ab.

78. individual variability.ti,ab.

79. (variability and (analysis or values)).ti,ab.

80. (uncertainty and (measurement or measuring)).ti,ab.

81. (standard error adj1 measurement).ti,ab.

82. (sensitiv* or responsive*).ti,ab.

83. ((minimal or minimally or clinical or clinically) and (important or significant or detectable) and (change or difference)).ti,ab.

84. (small* and (real or detectable) and (change or difference)).ti,ab.

85. meaningful change.ti,ab.

86. ceiling effect.ti,ab.

87. floor effect.ti,ab.

88. Item response model.ti,ab.

89. (IRT or Rasch or DIF).ti,ab.

90. Differential item functioning.ti,ab.

91. computer adaptive testing.ti,ab.

92. item bank.ti,ab.

93. cross-cultural equivalence.ti,ab.

94. or/55-93

95. 54 and 94
