# Supplementary material for: How best to assess quality of life in informal carers of people with dementia; A systematic review of existing outcome measures
Source: PLoS One. 2018 Mar 14;13(3):e0193398. doi: 10.1371/journal.pone.0193398 (PMC5851581; doi:10.1371/journal.pone.0193398)
Supplement: S3 File — (DOCX) [file pone.0193398.s003.docx]

# Excluded articles

## Articles not available in English

1. Abe K. Reliability and validity of the Subjective Well-Being Index for family caregivers. Japanese Journal of Health Psychology. 2004;17(1):47-55.(also uncertainty here regarding target population and construct)

2. Bagne BM, Gasparino RC. Quality of life of Alzheimer patients’ caregivers. Revista Enfermagem UERJ. 2014;22(2):258-63 6p.

3. Laicardi C, Pezzuti L. Development and validation of an evaluation of relatives rating scale. Archivio di Psicologia, Neurologia e Psichiatria. 1997;58(2-3):145-64.

4. Teresa Dominguez-Guedea M, Diaz-Loving R. Elder's Family Caregiver Abnegation Scale. Anales De Psicologia. 2016;32(1):224-33.

## Articles reporting on application of instrument only and not establishment of its measurement properties

1. Arango-Lasprilla JC, Lehan T, Drew A, Moreno A, Deng X, Lemos M. Health-Related Quality of Life in Caregivers of Individuals With Dementia From Colombia. American Journal of Alzheimers Disease and Other Dementias. 2010;25(7):556-61.

2. Banerjee S, Murray J, Foley B, Atkins L, Schneider J, Mann A. Predictors of institutionalisation in people with dementia. Journal of Neurology, Neurosurgery & Psychiatry. 2003;74(9):1315-6.

3. Black BS, Johnston DM, Rabins PV, Lyketsos CG, Samus QM. Quality of Life of Elders with Dementia and Their Caregivers: Use of Multiple Measures for Community-Based Translational Research. Quality of Life Research. 2010;19:26.

4. Bremer P, Cabrera E, Leino-Kilpi H, Lethin C, Saks K, Sutcliffe C, et al. Informal dementia care: Consequences for caregivers' health and health care use in 8 European countries. Health Policy. 2015;119(11):1459-71.

5. Coen RF, O'Boyle CA, Swanwick GRJ, Coakley D. Measuring the impact on relatives of caring for people with Alzheimer's disease: quality of life, burden and well-being. Psychology & Health. 1999;14(2):253-61 9p.

6. Gignac MA, Gottlieb BH. Caregivers' appraisals of efficacy in coping with dementia. Psychology & Aging. 1996;11(2):214-25.

7. Gusi N, Prieto J, Madruga M, Garcia JM, Gonzalez-Guerrero JL. Health-Related Quality of Life and Fitness of the Caregiver of Patient with Dementia. Medicine and Science in Sports and Exercise. 2009;41(6):1182-7.

8. Harwood DG, Barker WW, Ownby RL, Duara R. Caregiver self-rated health in Alzheimer's disease. Clinical Gerontologist. 2000;21(4):19-33 15p.

9. Hua Y, Yi J, Junxia C. Investigation on quality of life of direct relatives as dementia patients' caregivers and its related factors. Chinese Nursing Research. 2013;27(1A):24-7 4p.

10. Inouye K, Pedrazzani ES, Pavarini SCI, Toyoda CY. Perceived quality of life of elderly patients with dementia and family caregivers: evaluation and correlation. Revista Latino-Americana de Enfermagem (RLAE). 2009;17(2):187-93 7p.

11. Kuo L-M, Huang H-L, Hsu W-C, Shyu Y-IL. Health-Related Quality of Life and Self-Efficacy of Managing Behavior Problems for Family Caregivers of Vascular Dementia and Alzheimer's Disease Patients. Dementia & Geriatric Cognitive Disorders. 2014;38(5/6):310-20 11p.

12. Markowitz JS, Gutterman EM, Sadik K, Papadopoulos G. Health-related quality of life for caregivers of patients with Alzheimer disease. Alzheimer Disease & Associated Disorders. 2003;17(4):209-14.

13. Mayor MS, Ribeiro O, Paúl C. Satisfaction in dementia and stroke caregivers: a comparative study. Revista Latino-Americana de Enfermagem (RLAE). 2009;17(5):620-4 5p

14. Meeuwsen EJ, Melis RJ, Adang EM. Cost-effectiveness of post-diagnosis treatment in dementia coordinated by multidisciplinary memory clinics in comparison to treatment coordinated by general practitioners: an example of a pragmatic trial. J Nutr Health Aging. 2009;13.

15. Orgeta V, Orrell M, Hounsome B, Woods B, Team R. Self and carer perspectives of quality of life in dementia using the QoL-AD. International Journal of Geriatric Psychiatry. 2015;30(1):97-104.

16. Pinto MF, Barbosa DA, Ferreti CEL, de Souza LF, Fram DS, Belasco AGS. Quality of life among caregivers of elders with Alzheimer's disease. Acta Paulista de Enfermagem. 2009;22(5):652-7 6p.

16. Scholzel-Dorenbos C. Quality of life of patients with Alzheimer's disease and their caregivers: Schedule for the Evaluation of Individual Quality of Life (SEIQoL). Tijdschrift voor Gerontologie en Geriatrie. 2000;31(1):23-6.

17. Sequeira C. Difficulties, coping strategies, satisfaction and burden in informal Portuguese caregivers. Journal of Clinical Nursing. 2013;22(3/4):491-500 10p.

18. Thomas P, Chantoin-Merlet S, Hazif-Thomas C, Belmin J, Montagne B, Clément J-P, et al. Complaints of informal caregivers providing home care for dementia patients: the Pixel study. International journal of geriatric psychiatry. 2002;17(11):1034-47.

19. Thomas P, Lalloue F, Preux PM, Hazif-Thomas C, Pariel S, Inscale R, et al. Dementia patients caregivers quality of life: the PIXEL study. International Journal of Geriatric Psychiatry. 2006;21(1):50-6.

20. Drummond MF, Mohide EA, Tew M, Streiner DL, Pringle DM, Gilbert JR. Economic Evaluation of A Support Program for Caregivers of Demented Elderly. International Journal of Technology Assessment in Health Care. 1991;7(02):209-19.

## Incorrect target population e.g. person with dementia, carers of people with other conditions only or general population

1. Aguirre E, Kang S, Hoare Z, Edwards RT, Orrell M. How does the EQ-5D perform when measuring quality of life in dementia against two other dementia-specific outcome measures? Quality of Life Research. 2016;25(1):45-9.

2. Becker S, Kruse A, Schroder J, Seidl U. The Heidelberg instrument for the assessment of quality of life in people suffering from dementia (HILDE.) - dimensions of quality of life and methods of operationalization. Zeitschrift Fur Gerontologie Und Geriatrie. 2005;38(2):108-21.

3. Berg-Weger M, Rauch SM, Rubio DM, Tebb SS. Assessing the health of adult daughter former caregivers for elders with Alzheimer's disease. American journal of Alzheimer's disease and other dementias. 2003;18(4):231-9.

4. Bouman AIE, Ettema TP, Wetzels RB, van Beek APA, de Lange J, Dröes RM. Evaluation of Qualidem: a dementia-specific quality of life instrument for persons with dementia in residential settings; scalability and reliability of subscales in four Dutch field surveys. International Journal of Geriatric Psychiatry. 2011;26(7):711-22 12p.

5. Brod M, Stewart AL, Sands L, Walton P. Conceptualization and measurement of quality of life in dementia: the dementia quality of life instrument (DQoL). Gerontologist. 1999;39.

6. Brouwer WBF, van Exel NJA, van Gorp B, Redekop WK. The CarerQol instrument: A new instrument to measure care-related quality of life of informal caregivers for use in economic evaluations. Quality of Life Research. 2006;15(6):1005-21.

7. Bruce E. Looking after well-being: a tool for evaluation. Journal of Dementia Care. 2000;8(6):25-7 3p.

8. Buessing A, Fischer J, Haller A, Heusser P, Ostermann T, Matthiessen PF. VALIDATION OF THE BRIEF MULTIDIMENSIONAL LIFE SATISFACTION SCALE IN PATIENTS WITH CHRONIC DISEASES. European Journal of Medical Research. 2009;14(4):171-7

9. Buxton MJ, Lacey LA, Feagan BG, Niecko T, Miller DW, Townsend RJ. Mapping from disease-specific measures to utility: an analysis of the relationships between the Inflammatory Bowel Disease Questionnaire and Crohn’s Disease Activity Index in Crohn’s disease and measures of utility. Value Health. 2007;10.

10. Chia EM, Chia EM, Rochtchina E, Wang JJ, Mitchell P. Utility and validity of the self-administered SF-36: findings from an older population. Annals of the Academy of Medicine, Singapore. 2006;35(7):461-7.

11. Coucill W, Bryan S, Bentham P, Buckley A, Laight A. EQ-5D in patients with dementia: an investigation of inter-rater agreement. Med Care. 2001;39.

12. Dichter MN, Schwab CGG, Meyer G, Bartholomeyczik S, Dortmann O, Halek M. Measuring the quality of life in mild to very severe dementia: Testing the inter-rater and intra-rater reliability of the German version of the QUALIDEM. International Psychogeriatrics. 2014;26(5):825-36 12p.

13. Dourado MCN, Mograbi DC, Santos RL, Sousa MFB, Nogueira ML, Belfort T, et al. Awareness of Disease in Dementia: Factor Structure of the Assessment Scale of Psychosocial Impact of the Diagnosis of Dementia. Journal of Alzheimers Disease. 2014;41(3):947-56.

14. Edberg A-K, Anderson K, Orrung Wallin A, Bird M. The Development of the strain in dementia care scale (SDCS). International Psychogeriatrics. 2015;27(12):2017-30 14p.(professional caregivers only)

15. Ettema TP, Dröes RM, de Lange J, Mellenbergh GJ, Ribbe MW. QUALIDEM: development and evaluation of a dementia specific quality of life instrument. Scalability, reliability and internal structure. International Journal of Geriatric Psychiatry. 2007;22(6):549-56 8p.

16. Fanshel S, Bush JW. A Health-Status Index and its application to health-services outcomes. Oper Res. 1970;18.

17. Gerritsen DL, Ettema TP, Boelens E. Quality of life in dementia: do professional caregivers focus on the significant domains? Am J Alzheimers Dis Other Demen. 2007;22.

18. Glozman JM, Bicheva KG, Fedorova NV. Scale of quality of life of care-givers (SQLC). Journal of neurology. 1998;245(1):S39-S41.(carers of people with Parkinsons disease only)

19. Gomez-Gallego M, Gomez-Amor J, Gomez-Garcia J. [Validation of the Spanish version of the QoL-AD Scale in alzheimer disease patients, their carers, and health professionals]. Neurologia. 2012;27(1):4-10.

20. Hoeymans N, van Lindert H, Westert GP. The health status of the Dutch population as assessed by the EQ-6D. Qual Life Res. 2005;14.

21. Kasper JD, Black BS, Shore AD, Rabins PV. Evaluation of the Validity and Reliability of the Alzheimer Disease-related Quality of Life Assessment Instrument. Alzheimer Disease & Associated Disorders. 2009;23(3):275-84.

22. Kerner DN, Patterson TL, Grant I, Kaplan RM. Validity of the Quality of Well-Being Scale for patients with Alzheimer's disease. Journal of Aging & Health. 1998;10(1):44-61 18p.

23. Kirsch J, McGuire A. Establishing health state valuations for disease specific states: an example from heart disease. Health Econ. 2000;9.

24. Krabbe PF, Stouthard ME, Essink-Bot ML, Bonsel GJ. The effect of adding a cognitive dimension to the EuroQol multiattribute health-status classification system. J Clin Epidemiol. 1999;52.

25. León-Salas B, Logsdon RG, Olazarán J, Martínez-Martín P, The M-A. Psychometric properties of the Spanish QoL-AD with institutionalized dementia patients and their family caregivers in Spain. Aging & Mental Health. 2011;15(6):775-83 9p.

26. Logsdon RG, Gibbons LE, McCurry SM, Teri L. Assessing quality of life in older adults with cognitive impairment. Psychosom Med. 2002;64.

27. Lucas-Carrasco R, Gomez-Benito J, Rejas J, Brod M. The Spanish version of the dementia quality of life questionnaire: A validation study. Aging & Mental Health. 2011;15(4):482-9 8p.

28. Lucas-Carrasco R, Gomez-Benito J, Rejas J, Ott BR. The Cornell-Brown Scale for quality of life in dementia: Spanish adaptation and validation. Alzheimer Disease and Associated Disorders. 2013;27(1):44-50.

29. Lucas-Carrasco R, Lamping DL, Banerjee S, Rejas J, Smith SC, Gómez-Benito J. Validation of the Spanish version of the DEMQOL system. International Psychogeriatrics. 2010;22(4):589-97 9p.

30. Morris JC. Clinical dementia rating: a reliable and valid diagnostic and staging measure for dementia of the Alzheimer type. Int Psychogeriatr. 1997;9.

31. Mulhern B, Rowen D, Brazier J, Smith S, Romeo R, Tait R, et al. Development of DEMQOL-U and DEMQOL-PROXY-U: generation of preference-based indices from DEMQOL and DEMQOL-PROXY for use in economic evaluation. Health Technology Assessment (Winchester, England). 2013;17(5):v-xv, 1-140.

32. Mulhern B, Smith SC, Rowen D, Brazier JE, Knapp M, Lamping DL, et al. Improving the Measurement of QALYs in Dementia: Developing Patient- and Carer-Reported Health State Classification Systems Using Rasch Analysis. Value in Health. 2012;15(2):323-33 11p.

33. Naglie G, Tomlinson G, Tansey C. Utility-based quality of life measures in Alzheimer’s disease. Qual Life Res. 2006;15.

34. Novella JL, Ankri J, Morrone I, Guillemin F, Jolly D, Jochum C, et al. Evaluation of the quality of life in dementia with a generic quality of life questionnaire: The Duke Health Profile. Dementia and Geriatric Cognitive Disorders. 2001;12(2):158-66.

35. Novella JL, Jochum C, Ankri J, Morrone I, Jolly D, Blanchard F. Measuring general health status in dementia: practical and methodological issues in using the SF-36. Aging-Clinical & Experimental Research. 2001;13(5):362-9.

36. Peacock S, Misajon R, Iezzi A, Richardson J, Hawthorne G, Keeffe J. Vision and quality of life: development of methods for the VisQoL vision-related utility instrument. Ophthalmic Epidemiol. 2008;15.

37. Rabin R, de Charro F. EQ-5D: a measure of health status from the EuroQol Group. Ann Med. 2001;33.

38. Rabins PV, Kasper JD, Kleinman L, Black BS, Patrick DL. Concepts and methods in the development of the ADRQL: An instrument for assessing health-related quality of life in persons with Alzheimer's disease. Journal of Mental Health and Aging. 1999;5(1):33-48.

39. Ready RE, Ott BR. Integrating patient and informant reports on the Cornell-Brown quality-of-life scale. American Journal of Alzheimer's Disease & Other Dementias. 2007;22(6):528-34 7p.

40. Ready RE, Ott BR, Grace J. Factor structure of patient and caregiver ratings on the dementia quality of life instrument. Aging Neuropsychology and Cognition. 2007;14(2):144-54.

41. Ready RE, Ott BR, Grace J, Fernandez I. The Cornell-Brown Scale for Quality of Life in dementia. Alzheimer Disease and Associated Disorders. 2002;16(2):109-15.

42. Reisberg B, Ferris SH, de Leon MJ, Crook T. Global Deterioration Scale (GDS). Psychopharmacol Bull. 1988;24.

43. Rosas-Carrasco O, del Pilar Torres-Arreola L, de Guadalupe Guerra-Silla M, Torres-Castro S, Miguel Gutierrez-Robledo L. Validation of the Quality of Life in Alzheimer's Disease (QOL-AD) scale in Mexican patients with Alzheimer, vascular and mixed-type dementia. Revista De Neurologia. 2010;51(2):72-80.

44. Sanchez-Arenas R, Vargas-Alarcon G, Sanchez-Garcia S, Garcia-Peña C, Gutierrez-Gutierrez L, Grijalva I, et al. Value of EQ-5D in Mexican city older population with and without dementia (SADEM study). International Journal of Geriatric Psychiatry. 2014;29(5):478-88 11p.

45. Schmid R, Eschen A, Rueegger-Frey B, Martin M. Development and validation of the Inventory of Needs in Memory Impairment (BIG-65). Illness-related needs in people with cognitive impairment and dementia. Zeitschrift Fur Gerontologie Und Geriatrie. 2013;46(4):329-38.

45. Scholzel-Dorenbos CJM, Arons AMM, Wammes JJG, Rikkert MGMO, Krabbe PFM. Validation study of the prototype of a disease-specific index measure for health-related quality of life in dementia. Health and Quality of Life Outcomes. 2012;10.

46. Selai C, Vaughan A, Harvey RJ, Logsdon R. Using the QOL-AD in the UK. International Journal of Geriatric Psychiatry. 2001;16(5):537-8 2p.

47. Selai CE, Trimble MR, Rossor MN, Harvey RJ. Assessing quality of life in dementia: Preliminary psychometric testing of the Quality of Life Assessment Schedule (QOLAS). Neuropsychological Rehabilitation. 2001;11(3-4):219-43.

48. Smith SC, Lamping DL, Banerjee S, Harwood R, Foley B, Smith P, et al. Measurement of health-related quality of life for people with dementia: development of a new instrument (DEMQOL) and an evaluation of current methodology. Health Technology Assessment. 2005;9(10):1-+.

49. Smith SC, Lamping DL, Banerjee S, Harwood RH, Foley B, Smith P, et al. Development of a new measure of health-related quality of life for people with dementia: DEMQOL. Psychological Medicine. 2007;37(5):737-46.

50. Smith SC, Murray J, Banerjee S, Foley B, Cook JC, Lamping DL, et al. What constitutes health-related quality of life in dementia? Development of a conceptual framework for people with dementia and their carers. International Journal of Geriatric Psychiatry. 2005;20(9):889-95 7p.

51. Tatsumi H, Yamamoto M, Nakaaki S, Hadano K, Narumoto J. Utility of the Quality of Life-Alzheimer's Disease Scale for mild cognitive impairment. Psychiatry and Clinical Neurosciences. 2011;65(5):533.

52. Teunisse S, Derix MM. The interview for deterioration in daily living activities in dementia: agreement between primary and secondary caregivers. Int Psychogeriatr. 1997;9.

53. Ware JE. Sherbourne CD. The MOS 36-item short-form health survey (SF-36). I. Conceptual framework and item selection. Med Care. 1992;30.

54. Wasserman J, Aday LA, Begley CE, Ahn C, Lairson DR. Measuring health state preferences for hemophilia: development of a disease-specific utility instrument. Haemophilia. 2005;11.

55. Weitzner MA, Meyers CA, Steinbruecker S, Saleeba AK, Sandifer SD. Developing a care giver quality-of-life instrument. Preliminary steps. Cancer Practice. 1997;5(1):25-31. (caregivers of people with cancer only)

56. Wolak A, Jolly D, Drame M, Boyer F, Morrone I, Aquino JP, et al. Quality of life in dementia: Psychometric properties of a French language version of the Dementia Quality of Life questionnaire (DQoL). European Geriatric Medicine. 2010;1(6):334-47.

57. Wolak A, Novella J, Drame M, Guillemin F, Di Pollina L, Ankri J, et al. Transcultural adaptation and psychometric validation of a French-language version of the QoL-AD. Aging & Mental Health. 2009;13(4):593-600 8p.

58. Wolfs CAG, Dirksen CD, Kessels A, Willems DCM, Verhey FRJ, Severens JL. Performance of the EQ-5D and the EQ-5D+C in elderly patients with cognitive impairments. Health and Quality of Life Outcomes. 2007;5.

59. Yamamoto-Mitani N, Abe T, Okita Y, Hayashi K, Sugishita C, Kamata K. Development of a Japanese quality of life instrument for older adults experiencing dementia (QLDJ). International Journal of Aging & Human Development. 2002;55(1):71-95.

60. Yu H-M, He R-L, Ai Y-M, Liang R-F, Zhou L-Y. Reliability and validity of the Quality of Life-Alzheimer disease Chinese version. Journal of Geriatric Psychiatry and Neurology. 2013;26(4):230-6.

61. Hoefman RJ, van Exel J, Brouwer WBF. Measuring the impact of caregiving on informal carers: a construct validation study of the CarerQol instrument. Health and Quality of Life Outcomes. 2013;11.

62. Hoefman RJ, van Exel NJA, Foets M, Brouwer WBF. Sustained informal care: The feasibility, construct validity and test–retest reliability of the CarerQol-instrument to measure the impact of informal care in long-term care. Aging & mental health. 2011;15(8):1018-27.

63. Hoefman RJ, van Exel NJA, Looren de Jong S, Redekop WK, Brouwer WBF. A new test of the construct validity of the CarerQol instrument: measuring the impact of informal care giving. Quality of Life Research. 2011;20(6):875-87.

64. McKee KJ, Philp I, Lamura G, Prouskas C, Öberg B, Krevers B, et al. The COPE index--a first stage assessment of negative impact, positive value and quality of support of caregiving in informal carers of older people. Aging & Mental Health. 2003;7(1):39-52.

65. Balducci C, Mnich E, McKee KJ, Lamura G, Beckmann A, Krevers B, et al. Negative impact and positive value in caregiving: Validation of the COPE Index in a six-country sample of carers. The Gerontologist. 2008;48(3):276-86.

66. Rubio DM, Berg-Weger M, Tebb SS, Parnell LA. Comparing the well-being of post-caregivers and noncaregivers. American journal of Alzheimer's disease and other dementias. 2001;16(2):97-101.

67. Rubio DM, Berg-Weger M, Tebb SS, Rauch SM. Validating a measure across groups: The use of MIMIC models in scale development. Journal of social service research. 2003;29(3):53-67.

68. Logsdon RG, Gibbons LE, McCurry SM, Teri L. Quality of life in Alzheimer's disease: patient and caregiver reports. Journal of Mental Health and Aging. 1999;5:21-32.

## Article is not on establishment of measurement properties e.g. review article

1. Banerjee S, Willis R, Graham N, Gurland BJ. The Stroud/ADI dementia quality framework: a cross-national population-level framework for assessing the quality of life impacts of services and policies for people with dementia and their family carers. International Journal of Geriatric Psychiatry. 2010;25(3):249-57.

2. Cook DA, Beckman TJ. Current concepts in validity and reliability for psychometric instruments: theory and application. Am J Med. 2006;119.

3. Fritz CL, Farver TB, Kass PH, Hart LA. Correlation among three psychological scales used in research of caregivers for patients with Alzheimer's disease. Psychological Reports. 1997;80(1):67-80.

4. George LK, Gwyther LP. Caregiver well-being: A multidimensional examination of family caregivers of demented adults. The Gerontologist. 1986;26(3):253-9.

5. Glozman JM. Quality of life of caregivers. Neuropsychology review. 2004;14(4):183-96.

6. Hounsome N, Orrell M, Edwards RT. EQ-5D as a quality of life measure in people with dementia and their carers: Evidence and key issues. Value in Health. 2011;14(2):390-9.

7. Katona C, Livingston G, Cooper C, Ames D, Brodaty H, Chiu E. International Psychogeriatric Association consensus statement on defining and measuring treatment benefits in dementia. Int Psychogeriatr. 2007;19.

8. Krabbe PF. Thurstone scaling as a measurement method to quantify subjective health outcomes. Med Care. 2008;46.

9. Langenhoff BS, Krabbe PF, Wobbes T, Ruers TJ. Quality of life as an outcome measure in surgical oncology. Br J Surg. 2001;88.

10. Lenert LA, Rupnow MF, Elnitsky C. Application of a disease-specific mapping function to estimate utility gains with effective treatment of schizophrenia. Health Qual Life Outcomes. 2005;3.

11. Mosquera I, Vergara I, Larranaga I, Machon M, del Rio M, Calderon C. Measuring the impact of informal elderly caregiving: a systematic review of tools. Quality of Life Research. 2016;25(5):1059-92.

12. O'Connor CG. The development and testing of a model of Alzheimer's caregiving: Examination of racial and ethnic differences 2013.

13. Ozer S. QUALITY OF LIFE OF PATIENTS WITH DEMENTIA AND THEIR CAREGIVERS. Turkish Journal of Geriatrics-Turk Geriatri Dergisi. 2010;13:27-35.

14. Riepe MW, Mittendorf T, Forstl H. Quality of life as an outcome in Alzheimer’s disease and other dementias - obstacles and goals. BMC Neurol. 2009;9.

15. Rockwood K, Howlett S, Stadnyk K, Carver D, Powell C, Stolee P. Responsiveness of goal attainment scaling in a randomized controlled trial of comprehensive geriatric assessment. J Clin Epidemiol. 2003;56.

16. Schölzel-Dorenbos CJM, Krabbe PFM, Olde Rikkert MGM. Quality of life in dementia patients and their proxies; a narrative review of the concept and measurement scales. In: Preedy VR, Watson RR, editors. Handbook of Disease Burdens and Quality of Life Measures. Heidelberg, Germany: Springer; 2010.

17. Schölzel-Dorenbos CJM, Ettema TP, Bos J. Evaluating the outcome of interventions on quality of life in dementia: selection of the appropriate scale. Int J Geriatr Psychiatry. 2007;22.

18. Sheehan B. Assessment scales in dementia. Therapeutic Advances in Neurological Disorders. 2012;5(6):349-58.

18. Silberfeld M, Rueda S, Krahn M, Naglie G. Content validity for dementia of three generic preference based health related quality of life instruments. Quality of Life Research: An International Journal of Quality of Life Aspects of Treatment, Care & Rehabilitation. 2002;11(1):71-9.

19. Streiner DL, Norman GR. Health measurement scales: a practical guide to their development and use. Oxford: Oxford University Press; 2008.

20. Van Durme T, Macq J, Jeanmart C, Gobert M. Tools for measuring the impact of informal caregiving of the elderly: A literature review. International Journal of Nursing Studies. 2012;49(4):490-504

21. Vellone E, Piras G, Talucci C, Cohen MZ. Quality of life for caregivers of people with Alzheimer's disease. Journal of Advanced Nursing. 2008;61(2):222-31 10p.

22. Wilkin D, Mashiah T, Jolley DJ. Changes in behavioural characteristics of elderly populations of local authority homes and long-stay hospital wards, 1976–7. Br Med J. 1978;2.

Incorrect construct (e.g. burden, depression, psychological wellbeing, strain, grief, coping etc)

1. Al-Janabi H, Coast J, Flynn TN. What do people value when they provide unpaid care for an older person? A meta-ethnography with interview follow-up. Social Science & Medicine. 2008;67(1):111-21.

2. Al-Janabi H, Flynn TN, Coast J. Estimation of a Preference-Based Carer Experience Scale. Medical Decision Making. 2011;31(3):458-68.

3. Bachner YG, Ayalon L. Initial examination of the psychometric properties of the short Hebrew version of the Zarit Burden Interview. Aging & Mental Health. 2010;14(6):725-30.

4. Black SE, Gauthier S, Dalziel W, Keren R, Correia J, Hew H, et al. Canadian Alzheimer's disease caregiver survey: baby-boomer caregivers and burden of care. International Journal of Geriatric Psychiatry. 2010;25(8):807-13 7p

5. Branger C, O'Connell ME, Morgan DG. Factor analysis of the 12-item Zarit Burden Interview in caregivers of persons diagnosed with dementia. Journal of Applied Gerontology. 2016;35(5):489-507.

6. Braun M, Scholz U, Hornung R, Martin M. The burden of spousal caregiving: a preliminary psychometric evaluation of the German version of the Zarit burden interview. Aging & Mental Health. 2010;14(2):159-67.

7. Breinbauer K H, Vasquez V H, Mayanz S S, Guerra C, Millan K T. Original and abbreviated Zarit caregiver burden scales. Validation in Chile. Revista Medica De Chile. 2009;137(5):657-65.

8. Brogaard T, Neergaard MA, Guldin M-B, Sokolowski I, Vedsted P. Translation, adaptation and data quality of a Danish version of the Burden Scale for Family Caregivers. Scandinavian Journal of Caring Sciences. 2013;27(4):1018-26.

9. Coudin G, Mollard J. Difficulties, coping strategies and satisfactions in family caregivers of people with Alzheimer's disease: A first stage at validating the CADI-CAMI-CASI indices in a French sample. Geriatrie et Psychologie Neuropsychiatrie du Vieillissement. 2011;9(3):363-78.

10. de Boer AH, Oudijk D, Timmermans JM, Pot AM. [Self perceived burden from informal care: construction of the EDIZ-plus]. Tijdschrift voor Gerontologie en Geriatrie. 2012;43(2):77-88.

11. Erder MH, Wilcox TK, Chen WH, O'Quinn S, Setyawan J, Saxton J. A new measure of caregiver burden in Alzheimer's disease: the caregiver-perceived burden questionnaire. American Journal of Alzheimer's Disease & Other Dementias. 2012;27(7):474-82.

12. Gitlin LN, Winter L, Dennis MP, Hauck WW. Assessing perceived change in the well-being of family caregivers: psychometric properties of the Perceived Change Index and response patterns. American Journal of Alzheimer's Disease & Other Dementias. 2006;21(5):304-11.

13. Givens JL, Jones RN, Mazor KM, Prigerson HG, Mitchell SL. Development and Psychometric Properties of the Family Distress in Advanced Dementia Scale. Journal of the American Medical Directors Association. 2015;16(9):775-80.

14. Higginson IJ, Gao W, Jackson D, Murray J, Harding R. Short-form Zarit Caregiver Burden Interviews were valid in advanced conditions. Journal of Clinical Epidemiology. 2010;63(5):535-42.

15. Iecovich E. Psychometric properties of the Hebrew version of the Zarit Caregiver Burden Scale short version. Aging & Mental Health. 2012;16(2):254-63.

16. Jansen AP, van Hout HP, van Marwijk HW, Nijpels G, Gundy C, Vernooij-Dassen MJ, et al. Sense of Competence Questionnaire among informal caregivers of older adults with dementia symptoms: A psychometric evaluation. Clinical Practice and Epidemiology in Mental Health. 2007;3:11.

17. Jones PS, Winslow BW, Lee JW, Burns M, Zhang XE. Development of a Caregiver Empowerment Model to Promote Positive Outcomes. Journal of Family Nursing. 2011;17(1):11-28.

18. Kaufer DI, Cummings JL, Ketchel P. Validation of the NPI-Q, a brief clinical form of the Neuropsychiatric Inventory. J Neuropsychiatry Clin Neurosci. 2000;12.

19. Kinney JM, Stephens MAP. CAREGIVING HASSLES SCALE - ASSESSING THE DAILY HASSLES OF CARING FOR A FAMILY MEMBER WITH DEMENTIA. Gerontologist. 1989;29(3):328-32.

20. Kucukguclu O, Esen A, Yener G. The Reliability and Validity of The Caregiver Burden Inventory in Turkey. Journal of Neurological Sciences-Turkish. 2009;26(1):60-73.

21. Lawton MP. The Philadelphia Geriatric Center Morale Scale: a revision. J Gerontol. 1975;30

22. Lawton MP, Kleban MH, Moss M, Rovine M, Glicksman A. Measuring caregiving appraisal. Journal of Gerontology. 1989;44(3):P61-P71.

23. Liu H-Y, Wang Y-N, Huang H-L, Hsu W-C, Lin Y-E, Huang T-H, et al. Psychometric Properties of the Finding a Balance Scale for Family Caregivers of Elders with Dementia in Taiwan. Research in Nursing & Health. 2014;37(4):336-46.

24. Marwit SJ, Meuser TM. Development and initial validation of an inventory to assess grief in caregivers of persons with Alzheimer's disease. Gerontologist. 2002;42(6):751-65.

25. Marwit SJ, Meuser TM. Development of a short form inventory to assess grief in caregivers of dementia patients. Death Studies. 2005;29(3):191-205.

26. Mausbach BT, Roepke SK, Depp CA, Moore R, Patterson TL, Grant I. Integration of the pleasant events and activity restriction models: Development and validation of a "PEAR" model of negative outcomes in Alzheimer's caregivers. Behavior Therapy. 2011;42(1):78-88.

27. Montorio I, Losada A, Izal M, Marquez M. Dysfunctional thoughts about caregiving questionnaire: psychometric properties of a new measure. International Psychogeriatrics. 2009;21(5):913-21.

28. Moore PA. Development and evaluation of an Alzheimer's Caregiver Syndrome Scale (CSS) 2014.

29. Orgeta V, Lo Sterzo E, Orrell M. Assessing mental well-being in family carers of people with dementia using the Warwick-Edinburgh Mental Well-Being Scale. International Psychogeriatrics. 2013;25(9):1443-51.

30. O'Rourke N. Factor Structure of the Center for Epidemiologic Studies--Depression Scale (CES--D) Among Older Men and Women Who Provide Care to Persons with Dementia. International Journal of Testing. 2005;5(3):265-77.

31. Persson C, Wennman-Larsen A, Sundin K, Gustavsson P. Assessing informal caregivers' experiences: a qualitative and psychometric evaluation of the Caregiver Reaction Assessment Scale. European Journal of Cancer Care. 2008;17(2):189-99.

32. Riedijk S, Duivenvoorden H, Van Swieten J, Niermeijer M, Tibben A. Sense of competence in a Dutch sample of informal caregivers of frontotemporal dementia patients. Dementia and Geriatric Cognitive Disorders. 2009;27(4):337-43.

33. Romero-Moreno R, Marquez-Gonzalez M, Losada A, Gillanders D, Fernandez-Fernandez V. Cognitive fusion in dementia caregiving: Psychometric properties of the Spanish version of the "Cognitive Fusion Questionnaire". Behavioral Psychology / Psicologia Conductual: Revista Internacional Clinica y de la Salud. 2014;22(1):117-32.

34. Roth DL, Ackerman ML, Okonkwo OC, Burgio LD. The four-factor model of depressive symptoms in dementia caregivers: a structural equation model of ethnic differences. Psychology & Aging. 2008;23(3):567-76.

35. Schreiner A, Morimoto T, Arai Y, Zarit S. Assessing family caregiver's mental health using a statistically derived cut-off score for the Zarit Burden Interview. Aging & Mental Health. 2006;10(2):107-11.

36. Stevens AB, Coon D, Wisniewski S, Vance D, Arguelles S, Belle S, et al. Measurement of leisure time satisfaction in family caregivers. Aging & Mental Health. 2004;8(5):450-9.

37. Tan L, Yap P, Ng WY, Luo N. Exploring the use of the Dementia Management Strategies Scale in caregivers of persons with dementia in Singapore. Aging & Mental Health. 2013;17(8):935-41 7p.

38. Taub A, Andreoli SB, Bertolucci PH. Dementia caregiver burden: reliability of the Brazilian version of the Zarit caregiver burden interview. Cadernos de Saude Publica. 2004;20(2):372-6

39. Tennant R, Hiller L, Fishwick R, Platt S, Joseph S, Weich S, et al. The Warwick-Edinburgh Mental Well-being Scale (WEMWBS): development and UK validation. Health and Quality of Life Outcomes. 2007;5(1):1-13.

40. Vernooij-Dassen M, Kurz X, Scuvee-Moreau J, Dresse A. The measure of sense of competence in caregivers of patients with dementia. Revue D Epidemiologie Et De Sante Publique. 2003;51(2):227-35.

41. Volicer L, DeRuvo L, Hyer K, Piechniczek-Buczek J, Riordan ME. Development of a scale to measure quality of visits with relatives with dementia. Journal of the American Medical Directors Association. 2008;9(5):327-31.

42. Yu L, Insel KC, Reed PG, Crist JD. Family Caregiving of Older Chinese People With Dementia: Testing a Model. Nursing Research. 2012;61(1):39-50 12p.

43. Yu DSF, Kwok T, Choy J, Kavanagh DJ. Measuring the expressed emotion in Chinese family caregivers of persons with dementia: Validation of a Chinese version of the Family Attitude Scale. International Journal of Nursing Studies. 2016;55:50-9.

44. Goranitis I, Coast J, Al-Janabi H. An investigation into the construct validity of the Carer Experience Scale (CES). Quality of Life Research. 2014;23(6):1743-52.

45. Czaja SJ, Gitlin LN, Schulz R, Zhang S, Burgio LD, Stevens AB, et al. Development of the risk appraisal measure: a brief screen to identify risk areas and guide interventions for dementia caregivers. Journal of the American Geriatrics Society. 2009;57(6):1064-72.
