# Supplementary material for: How best to assess quality of life in informal carers of people with dementia; A systematic review of existing outcome measures
Source: PLoS One. 2018 Mar 14;13(3):e0193398. doi: 10.1371/journal.pone.0193398 (PMC5851581; doi:10.1371/journal.pone.0193398)
Supplement: S4 File — (DOCX) [file pone.0193398.s004.docx]

| **Instrument (and version)** | **Construct and Domains** | **1)Construct/Instrument Development**  **2) Target population** | **Number of:**  **i) scales**  **ii) items**  **iii) response categories** | **Scoring:**  **i) index or descriptive**  **ii) range** | **Administration**  **i) mode**  **ii) time** | **Instructions for completion** | **Summary of quality assessment for measurement properties** |
| --- | --- | --- | --- | --- | --- | --- | --- |
| ASCOT-Carer: INT4 version  (Rand et al.2015) | Social care-related quality of life (SCRQOL): the effect of social care support on quality of life.  7 domains: occupation, control over daily life, self-care, personal safety, social participation, space and time to be yourself, feeling supported and encouraged | 1) Domains identified by focus groups with informal carers and care managers (2007-2008). Instrument amended in 2012 following cognitive interviews with 31 informal carers (including 5 carers of people with dementia).  2) Informal carers of those in receipt of social services | i) 4  ii) 23  iii) 4 | i) index  ii) 0-21 | i) Interview (face to face or telephone)  ii) unknown | unknown | 1 study: fair (internal consistency, structural validity,  hypotheses testing) |
| Caregiver-targeted quality-of-life measure (CGQOL) (Vickrey et al. 2009) | CGQOL  10 dimension: caregiving assistance in ADLS, caregiving assistance in IADLS, personal time, role limitations due to caregiving, family involvement, demands of caregiving, worry, caregiver feelings, spirituality and faith, benefits of caregiving | 1) Focus groups (n=6) and cognitive interviews (n=29) with carers of people with dementia.  2) Informal carers of people with dementia | i) 10  ii) 80  iii) 5 | i)descriptive  ii) 0-100 | i) Telephone interview  ii) Median 23.5 minutes (IQR 18.5 – 30) | unknown | 1 study: fair (internal consistency, reliability and structural validity), excellent (hypotheses testing) |
| Carers of Older People in Europe (COPE) index  (Roud 2006) | Negative impact, positive value, quality of support | 1) 4 phases of development:  i) Theoretical framework developed by researchers following literature review  ii) double back translation to establish linguistic equivalence  iii) cross-cultural face validation: focus groups with expert and carer panel  iv) review of 3) by researchers: 5 additional items added  2) Informal carers of people aged ≥65 | i) 2 scales + 3 quality of support items + 1 question on financial difficulties  ii) 15  iii) 4 | i) descriptive  ii) unknown | i) Interview delivered by clinician  ii) 10-15 minutes | unknown | 1 study: Fair (internal consistency and hypotheses testing) |
| Caregiver Quality of Life Instrument (CQLI)  (Mohide et al. 1988) | 5 dimensions of wellbeing: amount of time to socialise with family and friends, quality of relationship with care recipient, degree of physical wellness and energy, adequacy of amount of sleep, degree of happiness and freedom from anxiety and frustration  Time trade-off technique: how many future years of life to trade-off for improved quality of life | 1) Dimensions identified by literature search and discussion with expert clinicians.  2) Informal carers of older people | i) 1  ii) 5 plus time trade-off tool  iii) 4 | i) index  ii) unknown | i) Trained interviewer (12h training) using highly structured interview schedule  ii) Mean 20 minutes (range 7 – 35) | Verbal and visual aids | 1 study: Fair (reliability),  Poor (hypotheses testing and responsiveness) |
| Carer well-being and support questionnaire (CWS)  (Quirk et al. 2012) | Carer Well-being (10 domains): your day-to-day life, your relationship with the person you care for, your relationships with family and friends, your financial situation, your physical health, your emotional wellbeing, stigma and discrimination, your own safety, the safety of the person you care for, your role as a carer. Carer Support scale (5 domains): information and advice for carers, your involvement in treatment and care planning, support from medical and/or care staff, support from other carers, taking a break (respite) | 1) Psychometric analysis of pre-existing instrument (CUES-C) and workshops with informal carers of people with mental health conditions including dementia.  2) Informal carers of people with mental health problems including dementia | i) 2  ii) CWS-v1: 74 (43 wellbeing, 31 support)  CWS-v2: 49 (32 wellbeing, 17 support)  iii) Wellbeing: 5  Support: 4 | i) descriptive  ii) unknown | i) Self-administered  ii) unknown | written | 1 study: CWS-v2: Excellent (internal consistency, content validity, structural validity, hypotheses testing), good (reliability) |
| Impact of Alzheimer’s Disease on Caregiver Questionnaire  (Cole et al. 2014) | Impacts of caregiving on HRQoL. 6 domains: emotional, physical, social, time, sleep and financial | 1) Draft instrument developed from systematic review; refined following focus group (21 informal carers of people with Alzheimer’s disease).  2) Informal carers of people with Alzheimer’s Disease | i) 1  ii) 12  iii) 5 | i) index  ii) 0 - 48 | i) Internet-based self-administered survey  ii) unknown | written | 1 study: Excellent (internal consistency), good (reliability), fair (hypotheses testing) |
| Quality of Life in Alzheimer’s Disease (QOL-AD) Questionnaire: Quality of life of the caregiver version (CQOL)  (Novelli et al. 2005, Novelli et al. 2010) | QOL-AD (Logsdon et al. 1999): appraisal of physical condition, mood, interpersonal relationships, ability to participate in meaningful activities, financial situation and overall assessment of self as whole and quality of life as a whole | 1) Items initially selected following review of literature on QOL in older adults and the assessment of QOL in other chronically ill populations. Items adjusted following review by people with Alzheimer’s disease and their caregivers, older adults with normal cognition and experts in geriatrics and gerontology. Interviewer instructions further amended after pilot study of 20 subjects.  2) Informal carers of people with Alzheimer’s disease | i) 1  ii) 13  iii) 4 | i) index  ii) 13-52 | i) Self-administered survey  ii) Mild dementia caregivers: 4.5±1.67 mins  Moderate dementia caregivers: 4.90±1.77 mins (Novelli et al. 2005) | written | 2 studies: Poor/fair (hypotheses testing, test-retest reliability, internal consistency and cross-cultural validity) |
| Major mediating and outcome variables in caring questionnaire (Schofield et al. 1997) | Major mediating and outcome variables in caring. Six domains: emotional well-being and physical health, family environment, caring role, help needed by care recipient, help provided by carer, behaviour problems in care recipient | 1) Existing instruments identified by review of literature, modified in some cases. Further items generated by exploratory individual and small group interviewers with informal caregivers.  2) Informal carers of people with congenital, traumatic and degenerative conditions (including dementia) living in the community | i) 19  ii) 159  iii) 5 for some, others verbal response coded by trained interviewer | i)descriptive  ii) unknown | i) Telephone interview administered by trained interviewer  ii) unknown | unknown | 1 study: Fair (internal consistency and structural validity) |
| Medical Outcomes Study Short-Form Health Survey (SF-36)  (Machniki et al. 2009) | Health-related Quality of Life (HRQoL)  Physical health subscales: physical functioning, role limitations due to physical health, bodily pain, general health perceptions  Mental health subscales: vitality, social functioning, role limitations due to emotional problems, general mental health. | 1) SF-20 instrument revised. Items added to domains for physical functioning, role functioning, bodily pain, social functioning and general health perception. Response choices for physical function revised. Items added to distinguish between role limitations due to physical and mental health problems. 5-item scale for general health perception revised.  2) General population | i) 8  ii) 36  iii) 3 | i) descriptive  ii) unknown | i) Self-administered questionnaire  ii) unknown | written | 1 study: Good (internal consistency, structural validity), fair (hypotheses testing) |
| Caregiver Well-Being Scale (first version, Tebb 1995) | Assesses well-being in terms of basic human needs and satisfaction with activities of daily living from a strength-based perspective. Basic human needs: expression of feelings, attendance to physical needs, security, self-esteem and esteem from others. Activities of daily living: time for self, household maintenance, leisure activities, maintenance of functions outside the home, family support | 1) Basic needs subscale (22 items) developed using Maslow’s (1968) hierarchy of needs. Development of activities of daily living subscale (23 items) guided by Weick’s (1986) health menu. Items examined for face validity by 4 individuals familiar with adult caregiving literature.  2) Informal caregivers of adults and children | i) 2  ii) 45  iii) 5 | i) descriptive  ii) unknown | i) Self-administered questionnaire  ii) unknown | written | 4 studies: excellent (internal consistency, content validity, structural validity), fair (hypotheses testing) |
| Caregiver Well-Being Scale (first version- refined by Rubio, Berg-Weber & Tebb 1999) | Compared to Tebb 1995 – factors in basic human needs reduced to: love, physical needs and self-security, and activities of daily living reduced to time for self, household maintenance and family | 1) As per first version (Tebb 1995)  2) All informal caregivers | i) 2  ii) 42 (3 items from basic human needs factor in Tebb’s 1995 questionnaire deleted due to poor reliability and validity)  iii) 5 | i) descriptive  ii) unknown | i) Self-administered questionnaire  ii) unknown | unknown |  |
| Caregiver Well-Being Scale (second version – Rubio et al. 2003) | Tebb’s 1995 version revised to 2 dimensions “Needs” and “Activities”. Based on Maslow’s (1962) Hierarchy of Needs: lower level needs (physiological needs must be met before higher level needs including (in order): need for safety, love and belongingness, self-esteem and self-actualisation. | 1) Items on original scale revised using Maslow’s (1962) hierarchy of needs. Content validity assessed by expert panel comprising 6 professionals and 6 lay experts (family caregivers of people with dementia).  2) Informal caregivers | i) 2  ii) 18  iii) 5 | i) descriptive  ii) unknown | Not applicable: assessment of content validity by expert panel | n/a |  |
| Caregiver Well-Being Scale: Short-Form Rapid Assessment (Tebb 2013) | Tebb’s 1995 version revised to 2 dimensions “Needs” and “Activities”. Based on Maslow’s (1962) Hierarchy of Needs: lower level needs (physiological needs must be met before higher level needs including (in order): need for safety, love and belongingness, self-esteem and self-actualisation. | 1) Original version revised using Maslow’s (1962) Hierarchy of Needs and results of earlier studies on its psychometric properties. Subscales renamed. Content validity assessed by expert panel (5 psychometricians and 1 social worker) and lay panel (10 family caregivers of people with Alzheimer’s disease): 11 items reworded and 1 item deleted.  2) Informal caregivers | i) 2  ii) 16 | descriptive | i) Self-administered questionnaire  ii) unknown | unknown |  |
