# Supplementary material for: How best to assess quality of life in informal carers of people with dementia; A systematic review of existing outcome measures
Source: PLoS One. 2018 Mar 14;13(3):e0193398. doi: 10.1371/journal.pone.0193398 (PMC5851581; doi:10.1371/journal.pone.0193398)
Supplement: S5 File — (DOCX) [file pone.0193398.s005.docx]

| **Instrument, (Country of Study)** | **Language of Instrument** | **Sample**  **i) size**  **ii) % female** | **Proportion of dementia carers in sample** |
| --- | --- | --- | --- |
| ASCOT-Carer (INT4 version)(UK) Rand et al.2015 | England | i) 387  ii) 58.9% | 39/387= 10% |
| CGQOL (USA) Vickrey et al. 2009 | English, Spanish | i) 200  ii) 79% | 100% |
| COPE index (New Zealand) Roud, Keeling and Sainsbury 2006 | English | i) 45  ii) 60% | 100% |
| CQLI (Canada) Mohide et al.1988 | English | i) 40  ii) 65% | 13/40 = 32.5%  (with carers of physically well older people and older people with physical impairments) |
| CWS (UK) Quirk et al. 2012 | English | i) Phase 1: 23  Phase 2: 210  Phase 3: 361  ii) Ph2 72%  Ph3 65.3% | Phase 1: 8/23= 34.7%  Phase 2 and 3:unknown; samples were carers of people with dementia or mental health problems |
| IADCQ (USA) Cole et al.2014) | English | i) 200  ii) 60% | 100% (carers of people with Alzheimer’s disease only) |
| QOL-AD: CQOL version (Brazil) Novelli et al. 2005, Novelli Nitrini & Caramelli 2010 | Portugese | Novelli et al. 2005  i) 40  ii) Mild dementia carers 80%,  moderate dementia carers 70% | 100% (carers of people with Alzheimer’s disease only) |
|  |  | Novelli Nitrini & Caramelli 2010  i) 60  ii) Mild dementia 80%, moderate dementia 66.7% | 100% (carers of people with Alzheimer’s only) |
| Major mediating and outcome variable in caring questionnaire (Australia) Schofield et al. 1997 | English | i) 976  ii) Unknown but female predominance noted | Unknown: diverse sample containing carers of people with congenital, traumatic and degenerative conditions |
| SF-36 (Argentina)Machniki et al. 2009 | Spanish | i) 52  ii) 85.4% | 100% carers of people with Alzheimer’s disease |
| Caregiver Well-Being Scale (USA) Tebb 1995,  Rubio, Berg-Weber & Tebb 1999, Rubio et al 2003, Tebb et al. 2013. | English | Tebb 1995, Rubio, Berg-Weber & Tebb 1999  i) 165  ii) 70% | 27/165 = 16.4%  Also included: 77 (46%) non-carers, 8 (5%) carers of children with severe developmental disabilities and 53 (32%) carers of “healthy” children <12 years old. |
|  |  | Rubio et al 2003  i) 12  ii) unknown | 50% - 6 lay experts on expert panel for content validity analysis (others were 5 academics engaged in research on family caregiving and one expert who worked with family caregivers) |
|  |  | Tebb et al. 2013  i) 493  ii) 96% | Informal carers from 3 other study samples:  1) 378 nurses, also informal carers  2) 100 carers of relatives with physical and/or cognitive impairment  3) 15 carers of relatives with dementia |
